# Metalloproteins in the Biology of Heterocysts

**DOI:** 10.3390/life9020032

**Published:** 2019-04-03

**Authors:** Rafael Pernil, Enrico Schleiff

**Affiliations:** 1 Institute for Molecular Biosciences, Goethe University Frankfurt, Max-von-Laue-Straβe 9, 60438 Frankfurt am Main, Germany; schleiff@bio.uni-frankfurt.de; 2 Frankfurt Institute for Advanced Studies, Ruth-Moufang-Straße 1, 60438 Frankfurt am Main, Germany; 3 Buchmann Institute for Molecular Life Sciences, Goethe University Frankfurt, Max-von-Laue-Straβe 15, 60438 Frankfurt am Main, Germany

**Keywords:** cyanobacteria, Nostocales, heterocysts, metalloproteins, metalloenzymes, metals, electron transport chains, oxidative stress, bioenergetics, metabolism

## Abstract

Cyanobacteria are photoautotrophic microorganisms present in almost all ecologically niches on Earth. They exist as single-cell or filamentous forms and the latter often contain specialized cells for N_2_ fixation known as heterocysts. Heterocysts arise from photosynthetic active vegetative cells by multiple morphological and physiological rearrangements including the absence of O_2_ evolution and CO_2_ fixation. The key function of this cell type is carried out by the metalloprotein complex known as nitrogenase. Additionally, many other important processes in heterocysts also depend on metalloproteins. This leads to a high metal demand exceeding the one of other bacteria in content and concentration during heterocyst development and in mature heterocysts. This review provides an overview on the current knowledge of the transition metals and metalloproteins required by heterocysts in heterocyst-forming cyanobacteria. It discusses the molecular, physiological, and physicochemical properties of metalloproteins involved in N_2_ fixation, H_2_ metabolism, electron transport chains, oxidative stress management, storage, energy metabolism, and metabolic networks in the diazotrophic filament. This provides a detailed and comprehensive picture on the heterocyst demands for Fe, Cu, Mo, Ni, Mn, V, and Zn as cofactors for metalloproteins and highlights the importance of such metalloproteins for the biology of cyanobacterial heterocysts.

## 1. Introduction

Proteins are involved in a broad spectrum of biological functions and catalyze a wide range of chemical reactions in cells [1]. However, the differences in the side chains of the twenty proteinogenic amino acids accounts for only a proportion of the chemical functionality of proteins found in nature. Incorporation of metal cofactors into their active sites further increases the functional diversity of the proteome. During evolution, different metals have been recruited for structural and catalytic roles on the basis of their chemical properties and natural availability. Such metal cofactors can be single or multiple metal atoms, clusters that contain metal and non-metal atoms, or small organometallic molecules [2,3]. They play a central role in protein function, structure, and stability [4,5,6]. Thus, removal of the metals or their replacement with other metals often leads to a drastic reduction or loss of protein activity [7]. Proteins with such cofactors are essential for the most complex and important biological processes, such as photosynthesis, respiration, transcription, and translation, as well as nitrogen fixation. This results from the participation of protein-bound metals in small molecule storage and transport, signal transduction, electron transfer, and chemical catalysis of numerous reactions [8,9,10].

The term *metalloprotein* is used to designate transient or permanent metal‒protein complexes that contain one or more metal atoms in their structure, either directly attached to the polypeptide chain or inserted into a non-protein organic molecule, which is bound subsequently to the polypeptide chain [2]. Metalloproteins are involved in structural and regulatory functions but also perform catalytic roles; they are then referred to as metalloenzymes and are represented in all six Enzyme Commission (EC) classes [11]. It is estimated that one third of all proteins in nature require metals to perform their biological roles and nearly half of all enzymes must associate with a particular metal to function [12,13]. This emphasizes the importance of metals in biology and highlights their remarkable role in conferring proteins with unique properties [14].

The dependence of all organisms on metalloproteins arises from the unique properties that metals confer to polypeptide chains. Metals in metalloproteins can form strong bonds that do not normally dissociate in biological conditions and, therefore, maintain the tertiary structure of proteins by linking amino acids that are widely separated in the polypeptide chain sequence [15]. Metals can also interact with more than one polypeptide chain and maintain the quaternary structure of oligomers to form active protein complexes [16,17,18]. Beside their structural role, metals are also required for the function of metalloproteins [19]. The dependence of metalloenzymes on metal cofactors originates from the inability of proteinogenic amino acid side chains to activate molecules like N_2_, H_2_, CH_4_, and CO, or their weakness in hydrolyzing simple but essential functional groups and compounds such as phosphates and peptides, respectively [20,21]. Specific functions catalyzed by metalloenzymes include (i) oxidation and reduction reactions, for which the most important metals are Fe, Mn, Cu, and Mo; (ii) radical-based rearrangement reactions that mainly require Fe or Co; (iii) methyl-group transfers catalyzed by Co; (iv) hydrolysis often involving Zn, Fe, Mg, Mn, or Ni; and (v) DNA regulation, generally requiring Zn [22].

In metalloproteins, an array of ligands is coordinated to a central metal atom or group of metals through coordinate covalent bonds [15,20,23,24]. The main ligands involved in metal binding are usually macrocyclic organic cofactors, such as heme, cobalamin, and chlorophyll, carbonyl and deprotonated amide groups of the peptide bonds present in the backbone of proteins [7,25], and N-, O-, and S-containing donor groups of the amino acid side chains [25]. The ligand groups in side chains that are found most often are imidazoles (His), thiolates (Cys), and carboxylates (Asp and Glu). Less frequently found ligands are amides (Asn and Gln), alcohols (Thr and Ser), thioethers (Met), and phenolates (Tyr), whereas aminos (Lys) and guanidines (Arg) serve only occasionally as ligands [7,15,26].

All arrays of ligands that are coordinated to a central metal atom or group of metals form metal coordination spheres. These spheres are fundamental for the function of metalloproteins, as they influence the selection of the appropriate metals, tune their properties, and optimize their reactivity [27]. However, not all metal ligands are provided by the polypeptide chain, amino acid residues, or organic cofactors. Solvents such as H_2_O and inorganic anions of low molecular weight, such as Cl^−^ and HCO_3_^−^, often contribute to complete the coordination spheres. Despite their importance, metal coordination complexes represent only a small part of the whole protein. Thus, it is the folded polypeptide chain that makes the function possible by constraining the geometry around the metal or by providing the appropriate pocket or environment for the substrate and/or product molecules [15].

**Figure 1:**
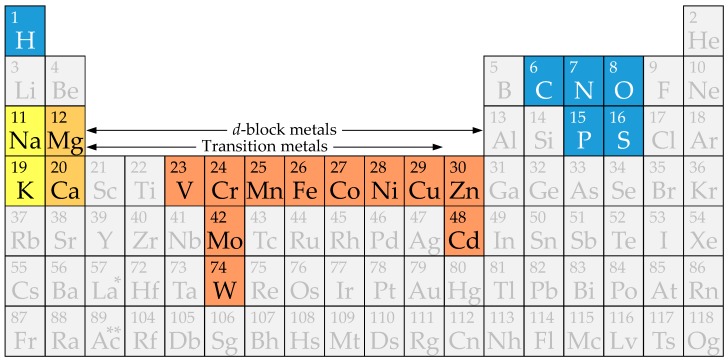
Biologically relevant metals found in organisms. Life is primarily based on the six bulk elements H, C, N, O, P, and S (blue), which are involved in basic biological organic chemistry. Alkali metals are highlighted in yellow, alkaline earth metals are shown in orange, and *d*-block metals, which include transition metals and group 12 metals (Zn and Cd), are presented in light red. *f*-block elements are not shown but their positions are indicated for lanthanides (_57_La*) and actinides (_89_Ac**).

The requirement for metals across the three domains of life is well established [4], although essential metals vary between organisms based on the energy and carbon metabolisms they perform. Moreover, some metabolic pathways are particularly metal-demanding, while others have lower or no metal requirements. Metals such as Na, K, Mg, Ca, Fe, Cu, Mn, Mo, and Zn are required by most organisms; others, such as Co, Ni, or V, are needed by specialized metalloenzymes in a smaller group of species. Finally, W, Sr, or Ba are known or suspected to have essential roles only in a few species [4,28,29]. The different metals contribute to the variety of functions of metalloproteins by their particular chemical properties [4]. Thus, biologically active metals are grouped in alkali metals (Na, K), alkaline earth metals (Mg, Ca), and *d*-block metals, which include transition metals (V, Cr, Mo, W, Mn, Fe, Co, Ni, Cu) and group 12 metals (Zn, Cd; Figure 1) [4,5,15,22,25]. Alkali and alkaline earth metals are present at high concentrations in organisms, while *d*-block metals are trace elements. 

The alkali metal ions Na^+^ and K^+^ exhibit labile interactions and bind weakly to organic ligands, have a high mobility in cells, and are important to generate ionic gradients across biological membranes and maintain osmotic balance (Table 1) [4,15,25]. K^+^ ions are also important for the activation of many enzymes. A prominent example is the glycolytic enzyme pyruvate kinase, where K^+^ ions are required to orient phosphoenolpyruvate in the substrate-binding pocket [25,30].

The alkaline earth metal ions Mg^2+^ and Ca^2+^ bind to organic ligands to some extent, have a moderate mobility in cells, and play structural and functional roles in metalloproteins [4,15,22,25]. Mg^2+^ is intimately associated with phosphate and is involved in numerous enzymatic phosphoryl-transfer reactions. Mg^2+^-dependent enzymes can interact directly with Mg^2+^ ions, which can modify the structure and/or play a catalytic role of such enzymes, or bind Mg^2+^‒substrate complexes, where the main interactions take place with the substrate. In photosynthetic organisms, Mg^2+^ is important as a metal center in light-absorbing chlorophylls [4,15,22,25]. Ca^2+^ is a charge carrier and it is important in cell signaling, regulation of key changes in cellular metabolism such as phosphorylation, dephosphorylation and transport, and activation of enzymes such as intra- and extracellular proteases (Table 1) [15].

The transition metals V, Cr, Mo, W, Mn, Fe, Co, Ni, and Cu bind tightly to organic ligands, have very little mobility in cells and exhibit multiple oxidation states, which make them ideal to participate in numerous redox reactions in metalloproteins [4,15,25]. Fe and Cu are essential because of their redox properties and are required in a vast range of metalloproteins, being involved in important biological processes such as photosynthesis and respiration [31,32,33,34]. Fe is present in the form of single- or multiple-atom centers, hemes, or Fe–S complexes [15,21,26,34,35,36]. It is involved in electron-transfer reactions and acid-base catalysis (Table 1), activation of O_2_ and other small molecules such as H_2_, CH_4_, and CO, transport of small compounds, and metal storage [4,15,21,25,26]. In photoautotrophic microorganisms such as cyanobacteria, the photosynthetic electron transport chain alone requires up to 24 atoms of Fe per set, contributing toward a quota of Fe 10 times greater than that of chemoheterotrophic microorganisms, such as *Escherichia coli* [37,38]. Remarkably, cyanobacteria also require higher Fe–C quotas than eukaryotic phytoplankton [39,40]. In turn, Cu is present in metalloproteins in the form of single- or multiple-atom centers and is often combined with other metals [34,35]. It is involved in a large number of electron-transfer and acid-base catalysis reactions (Table 1), the activation of O_2_ and also other gas molecules such as N_2_O, CH_4_, and CO, and transport of small molecules [4,15,22,25]. Cu is an important element in respiratory electron chains for terminal oxidases in photoautotrophic, photoheterotrophic, and chemoheterotrophic microorganisms and plays a relevant role in the photosynthetic apparatus of photoautotrophs [41]. 

Co and Ni are less common in metalloproteins, as their roles in acid-base catalysis are easily replaced by Zn, while their functions in redox catalysis can be substituted by Fe, Cu, or Mn [25]. However, Co and Ni are still required in some hydrolytic and oxidoreductase metalloenzymes [15]. Moreover, Co is vital in isomerization and methyl-transfer reactions, while Ni metalloenzymes are particularly important in the metabolism of small molecules like CO, H_2_, and CH_4_, although these enzymes are restricted to a limited number of organisms (Table 1) [4,15,22,25].

Mn is important because of its redox properties (Table 1). It plays an essential role in metalloenzymes involved in the detoxification of reactive O_2_ species such as superoxide dismutases, catalases, and peroxidases [46,47]. In cyanobacteria, Mn is demanded in high quantity by the H_2_O-splitting O_2_-evolving complex of the photosystem II. It donates electrons to the photosynthetic electron transport chain, making Mn requirements of cyanobacteria 100 times greater than that of photoheterotrophic microorganisms such as the purple bacterium *Rhodobacter capsulatus*, which performs anoxygenic photosynthesis [48]. Mn^2+^ is also a close, although not exact, Mg^2+^ surrogate and can be involved in many reactions catalyzed by the alkaline earth metal [25].

Mo is an essential metal of the N_2_-fixing enzyme nitrogenase and is also widely used in many redox enzymes (Table 1), especially in electron transfer reactions between one- and two-electron redox systems [4,15,22,25]. V has similar redox properties to those of Mo and is required in alternative nitrogenases by some microorganisms, including anaerobic bacteria but also cyanobacteria (Table 1), and other metalloenzymes in few organisms [4,25,49]. W exhibits similar chemical properties to those of Mo and is important in the metabolism of some thermophilic bacteria and hyperthermophilic archaea species that replace Mo with W (Table 1) [4,15,25].

Cr seems to be required by some organisms [45,50,51], but no Cr-dependent metalloproteins have been found in prokaryotes at present. In animals, Cr^3+^ ions bind to the peptide chromodulin, which functions as a Cr^3+^ carrier that could be involved in insulin signaling and sugar metabolism through a mechanism similar to that of Ca^2+^ signaling (Table 1) [45].

*d*-block, group 12 metals Zn and, to a much lesser extent, Cd, are not formally transition metals because they do not contain incomplete *d* sub-shells ([Ar]3*d*^10^4*s*^2^ and [Kr]4*d*^10^5*s*^2^, respectively) [4,15,22,25]. Both metals generally show the oxidation state +2 [22]. Zn^2+^ binds moderately to organic ligands, has intermediate mobility in cells and often plays a structural role, but is also involved in a number of catalytic reactions (Table 1). Zn^2+^ is the only metal ion required by metalloenzymes of all six EC classes, which illustrates its importance in catalysis and the versatility of its chemical properties [1]. Moreover, structural elements known as Zn fingers are universally found in the regulation of gene expression. Cyanobacteria also contain Zn-rich proteinaceous compartments called carboxysomes, which house rubisco and the Zn-containing carbonic anhydrase required to drive a CO_2_-concentration mechanism that is vital to increase CO_2_ fixation during photosynthesis [52,53]. Cd^2+^ is a toxic metal ion for most organisms, but it is essential in the chemistry of marine diatoms as the preferred metal cofactor for carbonic anhydrase rather than Zn^2+^ (Table 1) [12]. This is the only known example of a metalloenzyme that has evolved to use this chemical element.

## 2. Cyanobacteria and Heterocysts

Cyanobacteria are one of the largest, most ecologically diverse, and important groups of bacteria on Earth [54] and the only group of prokaryotes able to perform oxygenic photosynthesis [55]. They are ancient organisms that were essential for the development of the current oxygenic atmosphere and in the evolution of life, as they were the first organisms that developed oxygenic photosynthesis [56,57,58]. Their ecological success has favored a vast distribution in almost any natural habitat, including marine, fresh-water, terrestrial, and extremophile species, and some strains can establish symbiosis with fungi, plants, sponges, or protists [59,60]. Cyanobacteria play a relevant role in the carbon and nitrogen cycles, contributing to an important fraction of the primary productivity of oceans and to the maintenance of the biosphere balance [61,62]. Further, cyanobacteria have metal requirements often absent in other bacteria [28], which makes them highly metal-dependent and prolific organisms in the management and use of transition metals [28,55,63]. Besides the metal requirements in cyanobacteria already mentioned (see Section 1), the respective enzymes of reactions coupled to photosynthesis that assimilate inorganic carbon, nitrogen, and sulfur into organic molecules, along with other enzymes that are part of general metabolic pathways such as the glycolysis, the oxidative pentose phosphate pathway (OPPP), and the Krebs cycle, also contribute to additional metal demands in these organisms [63]. Thus, the reduction of nitrate and sulfate for N and S incorporation into amino acids exploits metalloenzymes that require uncommon cofactors, such as the Fe-containing siroheme in sulfite and nitrite reductases and the Mo-cofactor molybdopterin in nitrate reductase [64].

Cyanobacteria display a very diverse morphology, including rod- or coccus-shaped unicellular forms and filamentous species that show different degrees of filament complexity [65], but represent a coherent phylogenetic group [66,67]. Despite this morphological diversity, they exhibit a rather homogeneous metabolism and are primarily obligate photoautotrophs that perform oxygenic photosynthesis and fix CO_2_ via the reductive pentose phosphate pathway, yet sugars can support chemoheterotrophic and photoheterotrophic growth in some species [65,68]. Oxygenic photosynthesis requires the coordinated and consecutive action of two photosystems (PSII and PSI) to generate the high redox potential needed to extract electrons from H_2_O in parallel with O_2_ release [69] and to produce ATP and reducing equivalents in the form of NADPH or reduced ferredoxin (Fd), which are used afterwards to fix CO_2_ and, in diazotrophic cyanobacteria, also N_2_ [70]. This photoautotrophic metabolism distinguishes cyanobacteria from the so-called photosynthetic bacteria, which group green and purple bacteria and perform different types of anoxygenic photosynthesis [71].

Cyanobacteria bear a Gram-negative cellular envelope that is composed of an inner cytoplasmic membrane surrounded by an outer membrane, confining an intermembrane space, termed periplasm, that contains a peptidoglycan layer [72,73]. However, the cell wall in cyanobacteria has characteristics that resemble those of Gram-positive bacteria, such as a thick peptidoglycan layer [74]. Multicellular forms of cyanobacteria consist of filaments that can contain hundreds of vegetative cells [65]. Remarkably, filamentous species display a continuous outer membrane along the entire filament [75]. Some filamentous cyanobacteria can undergo cellular differentiation processes that take place as adaptive responses to environmental changes, exhibiting up to four different cell types, such as constitutive vegetative cells and differentiated heterocysts, akinetes, and hormogonial cells [70]. Vegetative cells perform the oxygenic photosynthesis and CO_2_ fixation and, in response to combined nitrogen deprivation, can differentiate into heterocysts. These singular cells confine the metalloenzyme nitrogenase and are specialized in N_2_ fixation. Thus, the diazotrophic filament in heterocyst-forming cyanobacteria represents a multicellular organism with a special and remarkable supracellular structure in bacteria [70]. The interdependence between vegetative cells and heterocysts is extremely close and the survival and proliferation of the diazotrophic filament relies on multiple nutritional, metabolic, and regulatory relationships between both cell types. The role of heterocysts lies in fixing N_2_ and providing vegetative cells with fixed nitrogen compounds, while the role of vegetative cells consists in performing photosynthesis to fix CO_2_ and providing heterocysts with reduced carbon compounds [76]. Some heterocyst-forming strains can also form spores (akinetes), while some heterocystous and non-heterocystous species can produce hormogonia, which are small motile filaments involved in dispersion and colonization roles. Thus, the various differentiated cells confer novel metabolic capabilities, environmental resistance, or motility upon filamentous cyanobacteria to exploit other nutrients, stand unfavorable conditions, or disperse the colony [65,72,77,78,79].

### 2.1. General Properties of Heterocysts

Heterocysts are cells specialized in N_2_ fixation in aerobiosis where the metalloenzyme nitrogenase is expressed [80,81,82,83]. They are terminally differentiated cells that neither divide nor revert to the vegetative state [84] and display structural and functional differences as compared to vegetative cells. These distinct properties create a micro-oxic environment, which is required for the protection and optimal functioning of the O_2_-sensitive N_2_ fixation machinery and optimize the cell metabolism, which is essential to increase the efficiency of the N_2_ fixation reaction [79].

The transformation of vegetative cells into heterocysts represents a unique feature in nature, since no other multicellular organism, prokaryote or eukaryote, has evolved specialized cells that undergo such drastic physiological and morphological changes to create a suitable environment for N_2_ fixation [85]. Heterocysts differentiate in response to combined nitrogen deficiency [86], although there are indications that changes in light or temperature conditions can also stimulate their formation [87]. In heterocyst-forming cyanobacteria from the family Nostocaceae, which groups the model strains *Anabaena* (also known as *Nostoc*) sp. PCC 7120, *Nostoc punctiforme* PCC 73102, and *Anabaena variabilis*, heterocysts are found at semiregular intervals along the filament with a frequency of one heterocyst every ~10–15 cells [79]. This one-dimensional developmental pattern is maintained during diazotrophic growth with the differentiation of new heterocysts at approximately equidistant positions between two existing heterocysts in the filament.

When cells detect combined nitrogen deficiency, the differentiation is initiated with the degradation of specific proteins [88,89]. This response further involves the mobilization of storage nitrogen products, such as cyanophycin granules [90] and phycobiliproteins [91]. This process of degradation of proteins and cyanophycin is associated to the synthesis of new proteins and is required for the reorganization of the biochemical machinery of the developing cell.

The morphological and physiological features of heterocysts are the consequence of a different gene expression program than that of vegetative cells. During heterocyst development, a sequential activation of multiple genes at early, intermediate, and final stages takes place. These genes encode regulatory proteins of the differentiation process such as the transcriptional regulators NtcA and HetR, proteins required for remodeling the cell morphology, and enzymes involved in the heterocyst-specific metabolism [76,79,87,92,93,94,95]. Thus, while some genes are expressed exclusively in developing heterocysts, others are expressed only when mature heterocysts are formed, such as the *nifHDK* operon, which encodes the structural genes of the nitrogenase complex [83], *fdxH*, which encodes a heterocyst-specific Fd [96], or the operon *hupLS*, which encodes an uptake hydrogenase [97]. Moreover, some sets of genes are solely expressed in vegetative cells, such as the *rbcLXS* operon, which encodes the key enzyme for CO_2_ fixation ribulose-1,5-bisphosphate carboxylase/oxygenase [83], since vegetative cells and heterocysts have different metabolic roles in the diazotrophic filament. A third class of genes are active in both cell types, such as *glnA*, which encodes glutamine synthetase (GS) and is required in the metabolism of both cell types [98,99,100].

### 2.2. Morphology of Heterocysts

The development of heterocysts involves structural changes in vegetative cells [76,85]. These changes include the deposition of a distinctive multilayer envelope outside the cell, the formation of tight, narrow cell junctions between heterocysts and adjacent vegetative cells, and the rearrangement of the intracytoplasmic membrane system [101].

The heterocyst envelope consists of an inner laminated layer composed of heterocyst-specific glycolipids that creates a permeability barrier for gases [101,102,103] and an outer thicker, homogeneous layer made of specific polysaccharides that apparently protects the glycolipid layer from physical damage [104,105,106]. Moreover, the thickness of both the laminated glycolipid layer and the homogeneous polysaccharide layer is modulated in response to the extracellular concentration of O_2_ [107]. However, the heterocyst envelope must have the optimum degree of permeability to allow the entry into the cell of an amount of N_2_ sufficiently high enough to be reduced by the nitrogenase complex and a quantity of O_2_ sufficiently low enough to be consumed by the heterocyst respiratory activity, in order to maintain the O_2_ concentration at minimum intracellular levels [108].

The area of contact between heterocysts and vegetative cells is reduced to a very narrow septum, where the deposition of the heterocyst envelope results in the formation of a kind of *neck* or thin channel to minimize the diffusion of O_2_ into heterocysts [109,110,111]. However, it has been also proposed that the heterocyst wall could be highly impermeable to gases in general and N_2_ would enter exclusively into heterocysts from adjacent vegetative cells through the septa by a regulated mechanism [112].

A change in the distribution and nature of the intracytoplasmic membranes also takes place during differentiation. Thus, the peripheral distribution of the thylakoid membranes in vegetative cells disappears, forming a reticulated membrane system in the cytoplasm of heterocysts known as honeycomb [101,109,113,114]. These membranes are located next to the septa and have a high concentration of respiratory enzymes [104,115]. Moreover, heterocysts present two large granules of the cyanophycin polymer which are located at the heterocyst poles adjacent to vegetative cells [116] and act as a dynamic nitrogen reservoir [117,118,119,120].

### 2.3. Physiology and Metabolic Adaptations of Heterocysts

Heterocyst differentiation involves a wide range of metabolic and physiological changes to turn new differentiated cells into efficient N_2_-fixing factories with low O_2_ levels [76,101]. These modifications include (i) the lack of PSII activity and, thus, the absence of O_2_ production by photolysis of H_2_O to keep a low O_2_ concentration [101,121,122,123], (ii) the absence of photosynthetic CO_2_ fixation to avoid the use of energy and reducing equivalents in cellular processes other than N_2_ fixation, (iii) the expression of an uptake hydrogenase to recover energy and reducing equivalents from H_2_ produced as a byproduct during N_2_ fixation, (iv) the expression of a suite of metalloenzymes that protect heterocysts against reactive O_2_ species, (v) a high respiratory rate that provides energy for the N_2_ fixation reaction and contributes to elimination of O_2_ traces that could enter into the cells, and (vi) the synthesis of the nitrogenase complex and its auxiliary proteins [76,79,124,125].

The micro-oxic conditions in heterocysts are partly due to the gas permeability barrier created by the heterocyst envelope, but also due to a high respiratory rate in heterocysts [101]. This intense respiratory metabolism is determined by a high activity through the OPPP and terminal respiratory oxidases. This represents, in addition to an important mechanism of protection of nitrogenase, a source of ATP and reducing equivalents for N_2_ fixation. In *Anabaena* sp. PCC 7120, two groups of *cox* genes have been described to encode heme-copper terminal respiratory oxidases in heterocysts [126,127,128]. Heterocysts have a similar or even higher respiratory rate to that of vegetative cells, despite the fact that the former represent a minority regarding the vegetative cells in diazotrophic filaments [106]. Moreover, to compensate for the inactivation of nitrogenase by residual traces of O_2_ in heterocysts, there is also a high expression of *nif* genes that allows a high synthesis of polypeptides that form and assemble the nitrogenase complex [76].

Mature heterocysts also have a reduced amount of photosynthetic pigments compared to vegetative cells, since there is no de novo synthesis of phycobiliproteins and the only ones present originate from parental vegetative cells [129]. However, they remain active and constitute the antenna that transfer energy to PSI [130], enabling a cyclic photophosphorylation to generate ATP in mature heterocysts [76]. A non-cyclic flow of electrons via the PSI can also take place from NAD(P)H, through respiratory dehydrogenase complexes, and from the byproduct H_2_ generated by nitrogenase, through heterocyst hydrogenases. Therefore, ATP and reducing equivalents generated through this route are also used in N_2_ fixation. Moreover, superoxide radicals and other reactive oxygen species are generated as a result of one-electron O_2_ reduction at the acceptor side of PSI, which are degraded by the action of a suite of metalloenzymes present in heterocysts, such as superoxide dismutases and peroxidases [76,131].

Heterocysts lack ribulose 1,5-bisphosphate carboxylase/oxygenase and phosphoribulokinase [83,132,133,134], key enzymes for CO_2_ fixation in the reductive pentose phosphate pathway [135]. This renders heterocysts photoheterotrophic and dependent on vegetative cells, which provide heterocysts with organic compounds to generate ATP and reducing equivalents for N_2_ fixation and carbon skeletons for the assimilation of fixed nitrogen [136]. Moreover, these metabolic modifications ensure that the energy and reducing equivalents generated in heterocysts are directed to the fixation of N_2_ rather than CO_2_. Thus, vegetative cells provide heterocysts with the sugar sucrose, which is then split by an invertase to produce glucose and fructose [137,138,139]. Both sugars are metabolized by the initial steps of the glycolysis pathway and ultimately oxidized through the OPPP [140]. For this purpose, heterocysts exhibit a high expression of the gene *zwf*, which encodes glucose-6-phosphate dehydrogenase, a key enzyme in the OPPP [104,140]. Ammonium resulting from the reduction of N_2_ is immediately incorporated into the amino acid Glu via the enzyme GS, whose activity is high in heterocysts, producing Gln in the first instance [141] and then other amino acids [142]. However, heterocysts lack the enzyme glutamine:2-oxoglutarate aminotransferase (GOGAT), which synthesize Glu from Gln and 2-oxoglutarate (2-OG) [141]. Thus, Glu and Gln are mutually exchanged between heterocysts and vegetative cells [143].

Heterocysts depend on a wide variety of metals to maintain their specific functions and cellular metabolism. N_2_ fixation requires a significant number of metalloproteins of the photosynthetic and respiratory metabolism, which have high Fe and Cu requirements [40,144,145,146], to provide energy and electrons to this essential biological process for the nitrogen cycle [144]. Nitrogenases contain 38 atoms of Fe and either two Mo or two V atoms [147,148]. Other metalloenzymes are crucial to manage efficiently reactive oxygen species generated during the metabolism of heterocysts, while some metalloproteins are especially important in Fe storage, which is the most required transition metal in heterocysts [40,144,149]. These sets of metalloproteins are aimed at creating the optimal working conditions for the central metabolic enzyme of heterocysts, the nitrogenase complex, but also associated hydrogenases required to metabolize efficiently the H_2_ byproduct generated during the N_2_ reduction reaction to recover reducing equivalents that, otherwise, would be wasted. Moreover, the uptake hydrogenase accounts for 12 atoms of Fe and one Ni atom [150], while the bidirectional hydrogenase requires 27 atoms of Fe and one Ni atom [151]. The biological relevance of the *d*-block metals Fe, Cu, Mo, Mn, Ni, V, and Zn in heterocysts and their use by metalloproteins will be discussed in the present review.

## 3. Metalloproteins in N_2_ Fixation and H_2_ Metabolism

N_2_ fixation and H_2_ evolution are closely linked processes in heterocysts [152]. These specialized cells possess several nitrogenases and hydrogenases, which contain metals in their active sites that are only rarely used elsewhere in nature, such as Mo or V in nitrogenases and Ni in hydrogenases.

### 3.1. Mo- and V-Dependent Nitrogenases

Nitrogenases are metalloenzymes that catalyze the reduction of atmospheric N_2_ into bioavailable NH_3_ under aerobic conditions. They are the only biological catalysts for such a reaction, being essential in the biogeochemical cycle of nitrogen [153,154,155,156,157]. This process requires the cleavage of the N_2_ triple bond, one of the strongest bonds in nature, through the interplay of complex metal cofactors. Three homologous nitrogenases have been identified in nature, which are classified based on the metals present at their cofactor sites as Mo, V, or Fe nitrogenases [155,158]. All nitrogenases are O_2_-sensitive metalloenzymes, but heterocysts represent micro-oxic chambers for the expression of such enzymes. However, heterocysts express only Mo- and V-containing nitrogenase (Table 2), the former being present in all heterocyst-forming cyanobacteria and the latter only in *Anabaena variabilis* and a few closely-related cyanobacterial strains, but not, for example, in *Anabaena* sp. PCC 7120 and *Nostoc punctiforme* PCC 73102 [159,160,161].

All nitrogenases consist of two proteins, termed dinitrogenase and dinitrogenase reductase [162,163,164]. The dinitrogenase reductase is also known as Fe protein and contains one [4Fe–4S] cluster and two ATP binding sites. In turn, the dinitrogenase is also called MoFe protein (in Mo nitrogenases), VFe protein (in V nitrogenases) or FeFe protein (in Fe nitrogenases) and houses an electron-transfer P cluster as well as the active-site metal cofactor FeMo-co, FeV-co, or FeFe-co, respectively [148]. All nitrogenases consume high amounts of ATP and reducing equivalents, thus, heterocysts keep the respiratory and, partially, the photosynthetic electron transport chains around the PSI to support photophosphorylation and reduction of Fd in order to provide nitrogenase enzymes with energy and electrons [85].

**Figure 2:**
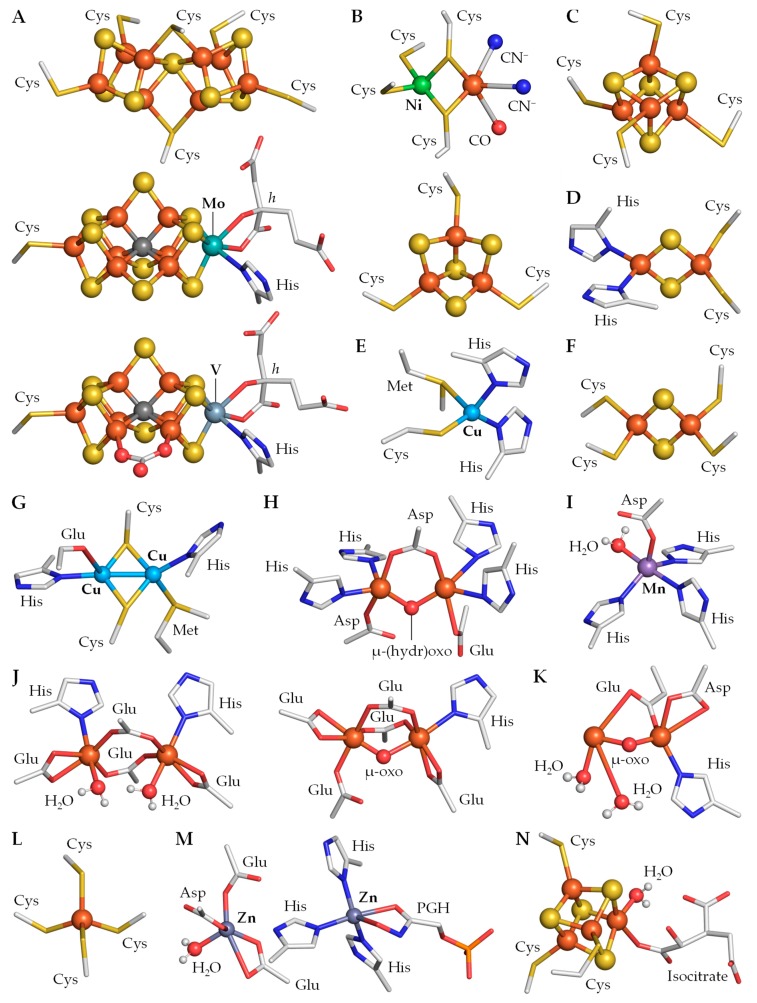
Representative metal clusters in heterocyst metalloproteins. (**A**) P cluster (top) and FeMo-co (middle; Mo nitrogenase, 4wza), and FeV-co (bottom; V nitrogenase, 5n6y). (**B**) Ni–Fe (top) and [3Fe–4S] (bottom) clusters (Ni–Fe hydrogenase, 3rgw). (**C**) [4Fe–4S] cluster F_B_ (photosystem I, 6hqb). (**D**) Rieske cofactor (cytochrome *b*_6_*f*, 4ogq). (**E**) Cu center (plastocyanin, 2cj3). (**F**), [2Fe–2S] cluster (ferredoxin FdxH, 1frd). (**G**) Cu–Cu center Cu_A_ (cytochrome *c* oxidase, 1qle). (**H**) Fe–Fe center (flavodiiron protein, 1ycf). (**I**) Mn center (Mn SOD, 1gv3). (**J**) reduced (left) and oxidized (right) Fe–Fe center (rubrerythrin, 1lko/1lkm). (**K**) Fe–Fe ferroxidase center (Dps protein, 1n1q). (**L**) [Fe–4S] cluster (rubrerythrin, 1lko). (**M**) Zn centers bound to the substrate analogue phosphoglycolohydroxamate (PGH; fructose-1,6-bisphosphate aldolase, 1b57). (**N**) catalytic [4Fe–4S] cluster bound to isocitrate (aconitase, 1b0j). *h*, homocitrate.

The Fe protein of the Mo nitrogenase is a γ_2_ homodimer encoded by *nifH* with a binding site for Mg^2+^-ATP provided by each subunit that serves as an ATP-dependent reductase in nitrogenase catalysis (Table 2) [154,165,166]. The two subunits of the Fe protein are bridged by a single [4Fe–4S] cluster through four Cys residues, two from each subunit, and electrons are delivered to this metal cluster in the first step [153]. In heterocysts, the NifH homodimer mediates the transfer of electrons from the electron donors Fd and flavodoxin to the MoFe protein [167,168]. Thus, flavodoxin and the enzyme pyruvate:ferredoxin (or flavodoxin) oxidoreductase (PFOR) NifJ (see Section 6) are required for N_2_ fixation under Fe-limiting conditions in *Anabaena* sp. PCC 7120 [169]. The cyanobacterial MoFe protein is an α_2_β_2_ heterotetramer encoded by *nifD* and *nifK*, respectively [153,166,170]. Each αβ heterodimer contains two unique metallo-sulfur clusters, namely the P cluster and the FeMo cofactor (FeMo-co), which is also known as M cluster (Table 2). The P cluster is an [8Fe–7S] cluster bridged between each αβ subunit pair by six Cys residues (Figure 2A, top), whereas the FeMo cofactor is a [Mo–7Fe–9S–C-homocitrate] cluster that is located within each α subunit and coordinated by one His and one Cys at opposite ends of the cluster (Figure 2A, middle) [153,156,171]. A remarkable feature in the structure of the FeMo cofactor is the presence of a central carbide coordinated to six atoms of Fe [172,173] that plays a structural function in stabilizing the active center of the Mo nitrogenase [174]. However, one cannot exclude a role of this C atom in regulating the reactivity of the metals in the M cluster [147].

The NifH homodimer undergoes a conformational rearrangement upon binding of two Mg^2+^-ATP molecules that enables the association with one αβ pair of the NifD_2_K_2_ tetramer and facilitates the inter-protein electron transfer from the former to the latter. This association initiates a series of events that result in transfer of an electron from NifH to the FeMo-co, hydrolysis of two ATP molecules into two ADP and two inorganic phosphates, phosphate release, and dissociation of NifH from NifDK. At this step, NifH is oxidized and contains two bound ADP, while NifDK is reduced by one electron [148,154]. Electrons are transferred sequentially from the [4Fe–4S] cluster of NifH to the M cluster of NifDK through the P cluster. This electron pathway illustrates the catalytic cooperation of the two protein components to reduce N_2_ within NifDK using successive electron equivalents provided by NifH [175]. The energy of the ATP hydrolysis is required for the dissociation of NifH and NifDK, but not for electron transfer. In turn, ATP binding is required to initiate a new cycle of N_2_ reduction [148,176]. This cycle is repeated to accumulate electrons in the active site, with four cycles needed to achieve the N_2_ binding state and eight cycles to complete the reduction of N_2_, the generation of one molecule of H_2_, and the release of the products [148]. The overall reaction catalyzed by the Mo nitrogenase is [147]:

N_2_ + 8*e*^−^ + 8H^+^ + 16Mg^2+^-ATP → 2NH_3_ + H_2_ + 16Mg^2+^-ADP + 16P*_i_*.

V and Mo nitrogenases share a good degree of similarity in the primary sequences and the metal cluster composition of their component proteins [155,158]. The Fe protein of the V nitrogenase is a γ_2_ homodimer-alike NifH and is encoded by *vnfH*. Moreover, it also has four conserved Cys ligands bridging the [4Fe–4S] cluster between both subunits, as well as a Mg^2+^-ATP binding site in each subunit (Table 2). Unlike MoFe dinitrogenases, VFe dinitrogenases are α_2_β_2_δ_2_ heterohexamers encoded by *vnfD*, *vnfK,* and *vnfG*, respectively [147,177]. The *vnfD*- and *vnfK*-encoded α and β subunits share some sequence similarity with the *nifD*- and *nifK*-encoded α and β subunits of the MoFe protein [158], while the *vnfG*-encoded δ subunit is unique [177]. The ligands for both the P and M clusters in the MoFe protein are also conserved in the sequence of the VFe protein [147]. Thus, in VFe dinitrogenases, the P cluster is an [8Fe–7S] moiety coordinated to six Cys residues between each αβ subunit pair, while the FeV cofactor (FeV-co; also known as V cluster) is a [V–7Fe–8S–C-homocitrate] cluster coordinated to a Cys and a His residue (Figure 2A, bottom; Table 2) [148,177].

V nitrogenases are less efficient during the ATP-dependent N_2_ reduction than Mo nitrogenases, consuming more energy and diverting a larger proportion of the electron flux from the Fe protein toward H_2_ formation during the reaction [152,178,179]. This leads to VFe nitrogenases acting purely as hydrogenases, exhibiting a specific activity for N_2_ fixation that is approximately 40% of that of MoFe nitrogenases, even under high N_2_ partial pressure [177]. Thus, VFe nitrogenases display a minimum observed reaction stoichiometry depicted as follows:

N_2_ + 12*e*^−^ + 12H^+^ + 40Mg^2+^-ATP → 2NH_3_ + 3H_2_ + 40Mg^2+^-ADP + 40P*_i_*.

The metal clusters in V nitrogenases exhibit distinct structural and redox features to those of Mo nitrogenases. NifH and VnfH are believed to contain identical [4Fe–4S] clusters. However, the Fe atoms in the cluster of VnfH exhibit a less ferric nature than that of the cluster of NifH [180]. Likewise, the P cluster of the VFe protein has long been regarded equivalent to its counterpart in the MoFe protein [181,182]. However, the P cluster in the VFe protein exists in a more oxidized state than its counterpart in NifDK in the resting state. Finally, the FeV cofactor (or V cluster) of the VFe protein is similar to the M cluster [147,181,183,184,185], but exhibits distinctive electronic structure and properties. These features originate from the chemical properties of V, the replacement of a S atom by a carbonate ester that bridges two atoms of Fe, and the interactions between the cofactors and their respective host proteins (Figure 2A) [147,148,186].

The V nitrogenase likely follows the same mode of action as the Mo enzyme during catalysis, forming a functional complex between the two components of the metalloenzyme to enable the ATP-dependent inter-protein transfer of electrons from the [4Fe–4S] center of the Fe protein, via the P cluster, to the FeV cofactor of the VFe protein for substrate reduction [147]. However, the unique structural features of the V nitrogenase may contribute to the less efficient N_2_ reduction catalysis of this nitrogenase, generating a lower NH_3_/H_2_ ratio than that of the Mo nitrogenase.

Whereas all heterocyst-forming cyanobacteria express Mo nitrogenases in heterocysts, *Anabaena variabilis* is unusual among them and exhibit two heterocyst nitrogenases, which are expressed based on the metal availability [160,161]. The primary nitrogenase is a Mo nitrogenase encoded by the *nif1* genes that is expressed when Mo is available [187,188]. However, when Mo is scarce but V is abundant, *A. variabilis* synthesizes the alternative V nitrogenase encoded by the *vnf* genes [187,189].

### 3.2. Hydrogenases

Hydrogenases catalyze the conversion of H_2_ to protons and electrons and, in some cases, also the reverse reaction to regenerate H_2_; they are found in archaea, bacteria, and some eukaryotes. These metalloenzymes are classified on the basis of the metal cluster present at their catalytic site as Ni–Fe, Ni–Fe–Se, Fe–Fe, and Fe hydrogenases [190]. However, heterocysts only harbor Ni–Fe hydrogenases.

The core of Ni–Fe hydrogenases is an αβ heterodimer that contains various metal clusters. The large α subunit houses a deeply buried binuclear Ni–Fe catalytic site, while the small β subunit contains up to three Fe–S clusters that, depending on the hydrogenase type, mediate the transfer of electrons from or to the Ni–Fe cluster [152]. In the large subunit, the binuclear Ni–Fe cluster is coordinated to four Cys residues of the protein and three unusual inorganic ligands such as two cyanide ions (CN^−^) and one carbon monoxide (CO; Figure 2B, top). The two metal atoms are held in close proximity via two disulfide bridges provided by two Cys residues, whereas the Ni atom is coordinated to the other two Cys residues and the three non-protein ligands are coordinated to the atom of Fe [191].

Heterocysts contain two distinct O_2_-sensitive Ni–Fe hydrogenases defined by their physiological role (Table 2). One is termed uptake hydrogenase, which is encoded by the *hupSL* operon and catalyzes the irreversible conversion of H_2_ into protons and electrons, while the second enzyme is a bidirectional hydrogenase, which catalyzes the reversible conversion of protons and electrons into H_2_ and is encoded by *hoxEFUYH* [152,192,193,194,195,196,197]. 

The uptake hydrogenase is an αβ heterodimer with a large *hupL*-encoded α subunit and a small *hupS*-encoded β subunit containing three metal clusters, namely one [3Fe–4S] and two [4Fe–4S] clusters (Table 2) [150,196,198]. The Fe–S cluster proximal to the [Ni–Fe] active site of the large subunit is a [4Fe–4S] cluster coordinated to one unusual Asn and three Cys residues [150]. It is electronically connected to a medial [3Fe–4S] cluster coordinated to three Cys residues (Figure 2B, bottom) and a distal [4Fe–4S] cluster coordinated to one uncommon Gln and three Cys residues [150,190,198]. Uptake hydrogenases are membrane-bound enzymes located on the cytoplasmic side of the thylakoid and plasma membranes [152,199], but they exhibit neither signal peptides nor transmembrane domains [195,200]. They likely interact with such membranes through a third membrane-embedded subunit or via interaction with the photosynthetic and/or respiratory electron transport chains [152,195], catalyzing in the [Ni–Fe] cluster the physiologically irreversible reaction:

H_2_ → 2H^+^ + 2*e*^−^.

Electrons generated in this reaction are transferred through the Fe–S clusters of HupS and directed probably to the plastoquinone (PQ) pools to form plastoquinol (PQH_2_) [152,195], being used then by the respiratory and photosynthetic electron transport chains in heterocysts. This mechanism enables heterocysts to recover ATP and reducing equivalents wasted as H_2_ during the nitrogenase catalysis [201,202], reduces the intracellular O_2_ levels to minimize nitrogenase inhibition [203,204,205], and prevents a high concentration of H_2_ to avoid negative effects on the nitrogenase reaction catalysis [152]. Thus, ATP and reductant recovered this way in heterocysts would be reused for N_2_ fixation and for other cellular processes [152]. Although electron entry from H_2_ through uptake hydrogenase to the PQ pools in the thylakoid and cytoplasmic membranes could be a key process for this H_2_ oxidation in the heterocyst metabolism, very little is known about the mechanism and factors involved in such a metabolic connection in heterocyst-forming cyanobacteria [152,206].

The bidirectional hydrogenase is a HoxEFUYH heteropentameric metalloenzyme and displays an αβ heterodimer [Ni–Fe] hydrogenase module that is formed by HoxYH, and a HoxEFU diaphorase moiety which serves as an enzymatic redox subcomplex (Table 2). HoxEFU couples the reversible cleavage of H_2_ to the oxidoreduction of low-potential electron carriers and is involved in electron transfer between such electron carriers and the Ni–Fe catalytic cluster [207,208]. In cyanobacteria, HoxEFU is considered a NAD(P)^+^/NAD(P)H-dependent enzyme, linking H_2_ uptake or evolution to NAD(P)H and NAD(P)^+^ as a source or sink for electrons respectively. It shows homology to the subunits NuoEFG of the respiratory Complex I of other bacteria and mitochondria [151,208,209]. Because some cyanobacteria that contain HoxEFU lack NuoEFG homologs, it has been speculated that HoxEFU might also be involved in respiration [209]. However, recent results have established Fd as the natural electron donor of the cyanobacterial bidirectional hydrogenase rather than the generally accepted NAD(P)H [210]. 

In the catalytic moiety, HoxH is the α subunit involved in the reaction catalysis and harbors the [Ni–Fe] active site. HoxY is the β subunit that coordinates a proximal [4Fe–4S] cluster to the [Ni–Fe] active site in the catalytic moiety and facilitates electron transfer to and from the hydrogenase active site [152,195,209,211,212]. This [4Fe–4S] cluster is coordinated to four putative Cys residues of the HoxY subunit and, unlike in HupS, is the only Fe–S cluster in this subunit. 

In the diaphorase module, HoxF harbors one [2Fe–2S] cluster and one [4Fe–4S] cluster and contains NAD(P)H/NAD(P)^+^ and FMN (flavin mononucleotide) binding motifs [152,209]. Each Fe–S cluster is coordinated to four putative Cys residues in this subunit [195,211,212]. HoxF is the large subunit of the diaphorase moiety and is involved in electron transfer from and to electron carriers. HoxU represents the small subunit and houses multiple Fe–S clusters in the form of one [2Fe–2S] and three [4Fe–4S] [209,212]. However, it is not clear whether one of these [4Fe–4S] clusters has a [4Fe–4S] or a [3Fe–4S] configuration [195,211]. Each Fe–S cluster is coordinated to four Cys residues in the protein subunit, but the uncertain [4Fe–4S] or [3Fe–4S] cluster is coordinated to a set of one His and three Cys residues or only to three Cys residues [195,211,212]. HoxU is involved in the electron transfer between the small subunit HoxY of the catalytic moiety and HoxF, thus connecting the electron flow between the deeply buried [Ni–Fe] catalytic site and the binding site for electron carriers on the surface of the hydrogenase complex. HoxE is a putative bridging subunit for membrane attachment that anchors the hydrogenase to the membrane [207,213,214]. This subunit contains a [2Fe–2S] cluster involved in electron transfer and is thought to couple the hydrogenase complex to the respiratory and photosynthetic electron transport chains on thylakoid and cytoplasmic membranes [152,209]. This Fe–S cluster is coordinated to four Cys residues in the protein subunit [211,212]. Given its similarities to subunits of the respiratory Complex I, the diaphorase moiety could interact with the respiratory complex NDH-1 (see Section 4), delivering electrons from NAD(P)H or receiving electrons to reduce NAD(P)^+^ [209].

The bidirectional hydrogenase is a soluble enzyme located in the cytoplasm that lacks membrane spanning domains [196,208], but it is associated with thylakoids and plasma membranes potentially through the HoxE subunit [211,215,216]. Thus, the hydrogenase complex could interact with the photosynthetic electron transport chain through an integral thylakoid membrane complex or with the respiratory electron transport chain through protein complexes located in the thylakoid and cytoplasmic membranes [152,192]. The bidirectional hydrogenase catalyzes the physiologically reversible reaction that interconverts protons and electrons with H_2_ gas from NAD(P)H or NAD(P)^+^ as the electron donor or acceptor as shown in the following reaction:

2H^+^ + NAD(P)H ⇆ H_2_ + NAD(P)^+^.

Despite the precise physiological role of the bidirectional Hox enzyme is still under debate, it is thought to function as an electron valve to release any excess electrons produced in the respiratory and PSI-dependent photosynthetic electron transport chains and other energy metabolic routes in heterocysts [209,210,215,217,218]. Therefore, this would represent a redox balancing mechanism to avoid the overreduction, and the consequent damage, of PSI and other complexes of the electron transport chains. Although the reversible hydrogenase in cyanobacteria has been considered a NAD(P)^+^/NAD(P)H-dependent enzyme, recent results show that it is a Fd-dependent enzyme in *Synechocystis* sp. PCC 6803 [192]. Thus, this suggests that bidirectional hydrogenases in cyanobacteria could actually be Fd-dependent enzymes, including the enzyme present in heterocysts. This would have important implications for the physiology of the heterocyst, where reduced Fd from PSI could donate electrons to the bidirectional hydrogenase as an electron valve to balance the redox status of the electron transport chains [192,210]. Moreover, the bidirectional hydrogenase could also receive electrons via Fd or flavodoxin generated by the enzyme PFOR.

## 4. Metalloproteins in Electron Transport Chains in Heterocysts

Heterocyst bioenergetics is based on a regulated and coordinated functioning of the respiratory and PSI-dependent photosynthetic electron transport chains and the supply of reduced carbon compounds in the form of sucrose and Ala from vegetative cells to provide heterocysts with reducing equivalents. These electron chains are composed of a series of metalloprotein complexes that transfer electrons from donors, such as NAD(P)H and Fd, to different acceptors via redox reactions performed by metal clusters. Thus, the electron transfer creates a proton electrochemical gradient that drives the synthesis of ATP and electrons are terminally accepted by O_2_ in the respiratory chain producing H_2_O, or Fd in the PSI-dependent photosynthetic chain.

In contrast, PSII is inactivated during heterocyst development (Table 3) [123]. By proteomic analysis of heterocysts isolated from *Nostoc punctiforme* ATCC 29133 or *Anabaena* sp. PCC 7120, a specific enrichment of one ATP-dependent Zn^2+^ protease of the FtsH family (Npun_R2022 and All3642, respectively; Table 3) has been observed [219,220]. Such proteases have been described to be involved in the degradation of, for example, the D1 protein of PSII [221] and, thus, are likely central metalloproteins for PSII inactivation and heterocyst development. Moreover, it has been suggested more recently that the PSII complex may remain intact in heterocysts and the inactivation process could involve the disruption of the supramolecular organization [123,222]. However, this mechanism of inactivation is not understood and remains largely unknown.

### 4.1. Photosynthetic Electron Transport Chain

In cyanobacteria, light-driven reactions of photosynthesis occur in thylakoid membranes and are mediated by PSI and PSII. PSI represents the largest photosynthetic requirement for Fe in the photosynthetic electron transport chain [37]. The coupling of the two reaction centers takes place in a linear electron transfer chain initiated by PSII from H_2_O and completed by PSI in the form of Fd and, ultimately, via the enzyme ferredoxin:NADP(H) oxidoreductase (FNR), NADPH. However, since heterocysts lack PSII activity, photosynthesis only operates through PSI [76,223].

**Figure 3:**
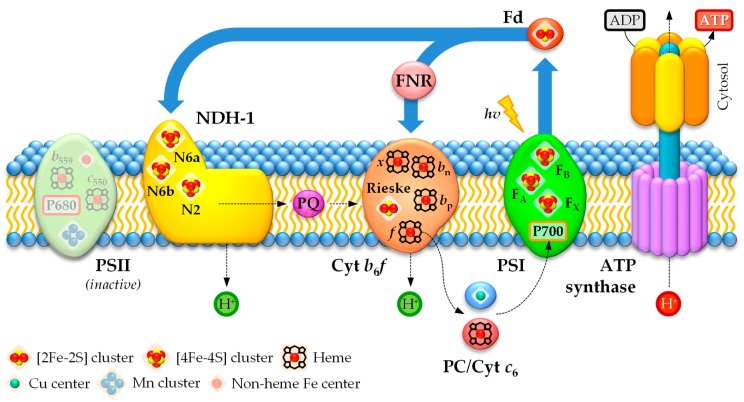
Metal requirements in the photosynthetic electron transport chain in heterocysts. Electrons can be transferred via three PSI-dependent (photosystem I-dependent) routes during photosynthesis. They can flow cyclically between the cytochrome *b*_6_*f* complex (Cyt *b*_6_*f*) and PSI via the soluble electron carriers plastocyanin (PC) or cytochrome *c*_6_ (Cyt *c*_6_) and the enzyme FNR (ferredoxin:NADP(H) oxidoreductase), which enable a flux of electrons from PSI-reduced ferredoxin (Fd) and Cyt *b*_6_*f*. Electrons can also be transferred linearly from the respiratory NDH-1 (type-I NAD(P)H dehydrogenase) complex to Cyt *b*_6_*f* via the plastoquinone (PQ) pool, before being transferred to PSI via PC or cytochrome *c*_6_. Fd is the final electron acceptor in the photosynthetic electron transport chain and is used as an electron donor for the nitrogenase or to form NADPH via FNR. Another cyclic route connects PSI and the NDH-1 complex via Fd. All cyclic and linear photosynthetic electron transport chains create a proton gradient across the membrane that is used by ATP synthase to produce ATP. Subunits of the NDH-1 complex, cytochrome *b*_6_*f* and PSI are not shown for the sake of simplicity.

In the photosynthetic electron transport chain in heterocysts, electrons can follow three pathways [85,123]. Böhme and colleagues proposed a cyclic electron transport between PSI and cytochrome *b*_6_*f* via the enzyme FNR to generate ATP (Figure 3), which supplies energy to nitrogenase and other metabolic processes [85,224,225]. However, a linear electron transport chain involving PSI and the NDH-1 (type-I NAD(P)H:plastoquinone oxidoreductase) complex of the respiratory chain (see Section 4.2.1), which functions as an electron source replacing PSII, has been proposed as the main pathway in heterocysts under light (Figure 3) [85,123]. Therefore, according to the linear electron transport pathway during PSI-dependent photosynthesis in heterocysts, electrons are transferred from the NDH-1 complex via the PQ pool to the cytochrome *b*_6_*f* complex, before being shuttled to PSI through the soluble electron carriers plastocyanin (PC) or cytochrome *c*_6_ [85,226]. Fd is the final electron acceptor in heterocysts in this linear photosynthetic electron transport chain and is used as an electron donor for nitrogenase or to generate NADPH [85,123]. Interestingly, since the cyanobacterial NDH-1 complex can use Fd as an electron donor (see Section 4.2.1), an additional cyclic photosynthetic electron transport chain via Fd → NDH-1 → PQ → cytochrome *b*_6_*f* → PC/cytochrome *c*_6_ → PSI → Fd could take place in heterocysts (Figure 3) [123]. Moreover, the soluble electron carriers PC and cytochrome *c*_6_ can also donate electrons to cytochrome *c* oxidase (see Section 4.2.2) [227]. However, recent results suggest that cytochrome *c*_6_ could be the main electron carrier in heterocysts [226]. 

The photosynthetic electron transport chain requires the participation of metal-rich protein complexes common to all photosynthetic organisms [85]. Cytochrome *b*_6_*f* contains six atoms of Fe, comprising the Rieske [2Fe–2S] cluster and four hemes (heme *f*, heme *b_p_*, heme *b_n_*, and heme *x*), and one Mg^2+^ ion bound to a chlorophyll *a* molecule [228]. The soluble electron carriers PC and cytochrome *c*_6_ contain one Cu and one Fe atom, respectively [229]. PSI contains 12 Fe atoms in the form of three [4Fe–4S] clusters and 96 Mg^2+^ ions bound to chlorophyll *a* molecules and Fd contains one [2Fe–2S] cluster [230].

#### 4.1.1. Photosystem I

PSI is a membrane metalloprotein complex that, in heterocysts, performs light-driven electron transfer from the luminal electron carrier PC (or cytochrome *c_6_* in Cu-deplete conditions) to the cytoplasmic, heterocyst-specific ferredoxin FdxH (Figure 3; see Section 4.1.3). Cyanobacterial PSI consists of nine transmembrane (PsaABFIJKLMX) and three cytoplasmic subunits (PsaCDE) and organizes in different oligomeric forms ranging from monomers to tetramers, depending on the species [85,229,231,232,233]. PSI proteins have been found in higher amounts in heterocysts than in vegetative cells, emphasizing the essential function of PSI in the bioenergetics of heterocysts [85,219,220,234]. In heterocysts in *Anabaena* sp. PCC 7120, PSI is present as tetramers [85]. Each PSI tetramer contains 48 atoms of Fe in 12 [4Fe–4S] clusters [229] and more than 25% of the cellular Fe quota in cyanobacteria is required for PSI exclusively [37]. Furthermore, each PSI monomer also binds 96 Mg^2+^-containing chlorophylls *a* (Table 3) [230].

PSI captures light energy by a large internal antenna system and guides it to the core of the reaction center (RC) with high efficiency. After excitation of the reaction center P700, the electron passes along the electron transfer chain consisting of the cofactors A_0_ (Chl *a*), A_1_ (phylloquinone), and the [4Fe–4S] clusters F_X_, F_A_, and F_B_ [235]. At the cytoplasmic side, the electron is donated by F_B_ to Fd and ultimately transferred to the enzyme FNR. Whereas the [4Fe–4S] cluster F_X_ is localized between subunits PsaA and PsaB and coordinated to two Cys residues from each subunit [236], PsaC harbors the [4Fe–4S] clusters F_A_ and F_B_ and exhibits a pseudo-two-fold symmetry similar to that of bacterial 2[4Fe–4S] ferredoxins (Table 3) [237]. However, PsaC also contains an extended loop connecting the Fe–S cluster-binding motifs and C- and N-terminal extensions that all together may be involved in docking Fd [237,238]. Both PsaC Fe–S clusters are coordinated to four Cys residues each from the protein subunit (Figure 2C for cluster F_B_) [239]. Thus, the arrangement of the clusters, with F_A_ being closer to F_X_ than F_B_, suggests an electron transfer sequence F_X_ → F_A_ → F_B_ → Fd (Figure 3) [235,237,238].

#### 4.1.2. Cytochrome b_6_f

PQH_2_ diffuses through the thylakoid membrane and docks onto the transmembrane cytochrome *b*_6_*f* complex [240]. At cytochrome *b*_6_*f*, electrons are transferred in the luminal side to the soluble electron carrier PC (or cytochrome *c*_6_ in Cu-deplete conditions), which migrates in the thylakoid lumen to dock onto the donor side of PSI and reduces its P700 reaction center (Figure 3). The final steps of the photosynthetic electron transport chain involve light absorption by PSI, photochemical conversion at its P700 reaction center, and electron transfer through the three PSI [4Fe–4S] clusters to the heterocyst electron carrier ferredoxin FdxH [228,241]. Ultimately, FdxH reduces the electron acceptor NADP^+^ to form NADPH via the enzyme FNR. Thus, the electron transfer along the photosynthetic electron transport chain in heterocysts generates a proton motive force across the thylakoid membrane, which is exploited by ATP synthase to produce ATP (Figure 3) [242]. ATP and reduced Fd, the products of the light-induced stages of the cyclic and linear (through the NDH-1 complex; see Section 4.2.1) photosynthesis in heterocysts, are then used for N_2_ fixation via the nitrogenase and for other important metabolic processes in heterocysts, previous synthesis of NADPH via FNR to supply some of them with an appropriate reducing power source.

The cytochrome *b*_6_*f* is a hetero-oligomeric integral-membrane protein complex that provides the electron connection between the NDH-1 complex and PSI in heterocysts (see Section 4.2.1) [243,244]. The cytochrome *b*_6_*f* complex is a functional dimer and each monomer contains eight polypeptide subunits. PetABCD are large subunits and confine the redox metal-containing cofactors heme *f* (*c*-type cytochrome *f*, PetA), hemes *b*_p_, *b*_n_, and *x* (cytochrome *b*_6_ and subunit IV, PetBD), and the Rieske [2Fe–2S] protein (PetC) (Figure 3; Table 3) [228]. The remaining four one-transmembrane-helix, small hydrophobic subunits are PetGLMN, which surround the core of the large subunits. In cytochrome *f*, the *c*-type heme *f* is covalently bound to the protein via two thioether linkages to Cys residues and the Fe atom is also coordinated to one His and an unusual N-terminal Tyr residue through its α-amino group as axial ligands [245,246,247], whereas the Fe atoms in the *b*-type hemes *b*_p_ and *b*_n_ in cytochrome *b*_6_ are coordinated to two His residues each as axial ligands [241,248]. Heme *x* is linked to the protein via a single thioether bond to a Cys residue; its Fe atom does not have axial protein ligands, but an H_2_O or OH^−^, which is unique for heme proteins [241,244,249]. Moreover, the Rieske protein exhibits a unique coordination of the [2Fe–2S] cluster through two His and two Cys residues (Figure 2D) rather than the standard coordination of [2Fe–2S] clusters via four Cys residues (see Figure 2F for comparison) [244,249]. An electron donated by PQH_2_ is transferred to the Rieske [2Fe–2S] protein and then to the heme *f* in the cytochrome *f*, which is involved ultimately in electron transfer to PC or cytochrome *c*_6_ in the thylakoid lumen. PQH_2_ also donates one electron to cytochrome *b*_6_ in the Q cycle, where the electron passes along the transport chain consisting of the metal-containing prosthetic groups heme *b*_p_ and heme *b*_n_ to ultimately reduce PQ. Both electron routes inside the cytochrome *b*_6_*f* also generate a proton electrochemical gradient across the thylakoid membrane, which is exploited to synthesize ATP through the ATP synthase complex (Figure 3). Although the role of heme *x* remains enigmatic to the present day, based on structural and redox potential data, it has been proposed to also be involved in the traditional Q cycle through the electron transfer sequence *b*_n_ → *x* → PQ and, more interestingly, in the Fd-dependent cyclic photosynthetic electron transport chain through the electron transfer sequence Fd → *x* → PQ [228,250].

#### 4.1.3. Soluble Electron Carriers

In addition to PSI and cytochrome *b*_6_*f*, the photosynthetic electron transport chain in heterocysts also requires metal-dependent small, mobile electron carriers to operate, such as PC, cytochrome *c*_6_, and Fd (Figure 3; Table 3) [226]. PC and cytochrome *c*_6_ act as one-electron shuttles between cytochrome *b*_6_*f* and PSI in the photosynthetic electron transport chain and between cytochrome *b*_6_*f* and terminal cytochrome oxidases in the respiratory electron transport chain and functionally link all complexes together [251]. Whereas PC contains one Cu atom, cytochrome *c*_6_ is an Fe-requiring metalloprotein and contains one heme group [145]. 

In *petE*-encoded PC, the Cu atom is coordinated to a Cys, a Met, and two His residues (Figure 2E) and the Cu site is located in a hydrophobic pocket in one side of the protein near the surface [252], which facilitates the transfer of electrons. Cytochrome *c*_6_ is a small protein encoded by *petJ* and harbors a *c*-type heme that is covalently attached through two thioether bonds to Cys residues. The coordination sphere of the Fe atom is completed by a His and a Met that act as axial ligands [253] and the heme group is located in a cleft at the surface of the protein. Cytochrome *c*_6_ plays similar roles to PC although it is expressed in Cu deficiency [226]. Ferredoxins are small, mostly acidic soluble proteins that exhibit a highly negative redox potential and harbor Fe–S clusters to deliver electrons to various metabolic pathways [254]. Ferredoxins taking part in photosynthetic reactions belong to the sub-class called plant-type ferredoxins, which are characterized by a [2Fe–2S] cluster, such as the cyanobacterial ferredoxins PetF and FdxH [255].

PetF is the main Fd in vegetative cells [85], whereas heterocysts contain the ferredoxin FdxH. As in plant-type [2Fe–2S] ferredoxins, the [2Fe–2S] cluster in the heterocyst-specific FdxH is coordinated to four Cys residues (Figure 2F). It is located at the outer edge of the protein in a loop region near the surface to facilitate the transfer of electrons [256]. FdxH receives electrons from the [4Fe–4S] cluster F_B_ in PSI under light, but can also be reduced by FNR in darkness with the use of NADPH produced in the OPPP [257]. FdxH is required for essential redox processes in heterocysts as an electron donor for N_2_ fixation delivering electrons to nitrogenase, cyclic photophosphorylation, and biosynthesis of chlorophyll [254,258,259]. In addition to FdxH, heterocysts contain a bacterial-type Fd termed FdxN. The structure of this Fd has not been determined and its role in heterocysts remains unclear, but it could be involved in the maturation of the nitrogenase complex, as it is the case with NifB-linked FdxN ferredoxins in other N_2_-fixing organisms [260,261]. In these organisms, FdxN displays two Cys-rich, binding motifs for Fe–S clusters, which are present in the cyanobacterial FdxN, and harbors two [4Fe–4S] clusters, each coordinated to four Cys residues [260,261,262]. Thus, it is assumed that FdxN in filamentous heterocyst-forming cyanobacteria also houses two [4Fe–4S] clusters [262].

Heterocysts have a very active Fd- and PSI-dependent cyclic photophosphorylation that generates ATP for N_2_ fixation [152]. Moreover, FNR catalyzes the Fd-dependent oxidation of NADPH derived from the OPPP. It has been proposed that the heterocyst ferredoxin FdxH is optimized for reverse electron flow between NADPH and FNR as compared to vegetative cells, so that Fd is eventually reduced from NADPH [263,264]. The key may lie in the slightly different redox potentials of PetF and FdxH. Thus, while PetF exhibits a redox potential of −380 to −390 mV, FdxH has a lower redox potential set at ~ −350 mV, which could be the critical feature in directing electrons from NADPH to Fd [85]. Despite such differences, there is a long debate about the reversibility of the Fd-NADP(H) redox reaction in heterocysts, because NADP(H) redox potential is set at −320 mV and the reduction of Fd from NADPH via FNR has been considered thermodynamically unfavorable [85,152]. Thus, this process is considered possible only when the ratio of NADPH to NADP^+^ is high, as measured in isolated heterocysts under N_2_ fixing conditions [265]. However, it has been shown more recently that this process is reversible in vivo in any condition, even in vegetative cells, and forward and reverse reactions via FNR might only have different catalysis mechanisms [266]. This has remarkable implications in the physiology of heterocysts. Thus, reduced FNR from OPPP-derived NADPH can transfer electrons directly to FdxH or to PSI through the cytochrome *b*_6_*f* complex and, ultimately, to FdxH, which acts as the intermediate electron donor to nitrogenase [224,258,267]. However, other ferredoxins yet to be discovered in heterocysts could also play an important role in this pathway [259].

### 4.2. Respiratory Electron Transport Chain

**Figure 4:**
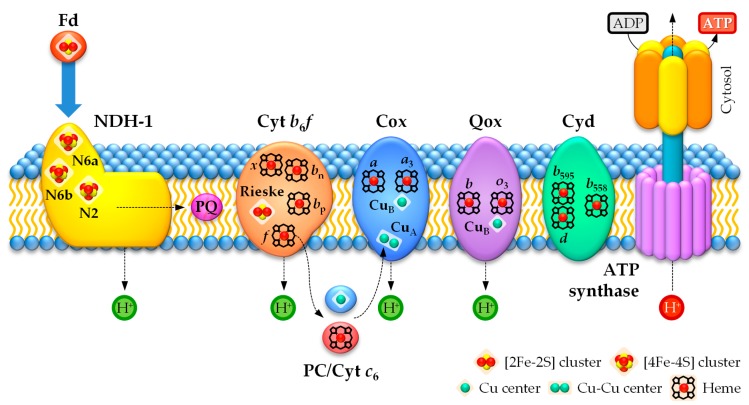
Metal requirements in the respiratory electron transport chain in heterocysts. During respiration, electrons are transferred from the NDH-1 complex to the cytochrome *b*_6_*f* complex (Cyt *b*_6_*f*) through the plastoquinone (PQ) pool, before being shuttled to the cytochrome *c* oxidase (Cox) via plastocyanin (PC) or cytochrome *c*_6_. In heterocysts, the alternative respiratory terminal oxidases quinol oxidase (Qox) and cytochrome *bd* quinol oxidase (Cyd) accept electrons from PQH_2_ (plastoquinol) and reduce O_2_. The respiratory electron transport chain generates a proton gradient across the membrane that is used by ATP synthase to produce ATP. Subunits of the NDH-1 complex, cytochrome *b*_6_*f,* and respiratory terminal oxidases are not shown for the sake of simplicity.

In contrast to the photosynthetic electron transport chain, the respiratory electron transport chain in cyanobacteria, and especially in heterocysts, is poorly understood. The main respiratory electron transport complexes in heterocysts include (i) a type-I NAD(P)H dehydrogenase (NDH-1) in the thylakoid membranes, (ii) terminal oxidases, such as a cytochrome *c* oxidase, (iii) alternative oxidases, and (iv) several components that are shared with the photosynthetic electron transport chain, such as the PQ/PQH_2_ pools, the cytochrome *b*_6_*f,* and the soluble electron carriers PC and cytochrome *c*_6_ (Figure 3 and Figure 4; Table 3 and Table 4) [85]. However, there is no evidence for the presence of a type-II NAD(P)H dehydrogenase (NDH-2) [268,269] or the Krebs cycle enzyme succinate dehydrogenase (SDH) in heterocysts. Regarding the latter, it is known that its competitive inhibitor malonate is ineffective in inhibiting the nitrogenase activity in heterocyst-forming cyanobacteria [270].

In heterocysts, NDH-1 is considered the principal respiratory electron donor protein complex [85], which is structurally and functionally similar to the mitochondrial respiratory complex I. It plays key roles both in respiration and cyclic photosynthetic electron flow around PSI [85,271]. Electrons from respiratory substrates enter the electron transport chain via PQ reduction by NDH-1 and are passed through cytochrome *b*_6_*f* and the luminal electron carriers PC or cytochrome *c*_6_ (Figure 4). However, unlike proteobacteria and mitochondria, cyanobacteria do not contain the cytochrome *bc*_1_ complex, and the cytochrome *b*_6_*f* is shared instead between the photosynthetic and respiratory electron transport chains (see Section 4.1.2) [272,273]. Electrons from PC or cytochrome *c*_6_ are subsequently transferred to either a terminal oxidase (Figure 4), which catalyzes conventional respiratory electron transport via O_2_ reduction into H_2_O, or PSI, which participates in a cyclic or linear photosynthetic electron transport chain (Figure 3). Both the full respiratory electron transport and the cyclic photosynthesis electron transport chains enable the synthesis of ATP, while the linear electron transport chain via PSI represents a route for both ATP synthesis and reduction of Fd. Thus, the redox state of the PQ pool plays an important role in steering the electron flow into different pathways, enabling the chain to adjust to different cellular and metabolic requirements [85,268,269,274,275]. In addition to respiration, multiple respiratory protein complexes also play a key role in photoprotection, allowing cyanobacteria to accommodate light fluctuations to prevent over-reduction of the interlinked electron transport chain with potential damaging consequences [269,274,276,277,278].

#### 4.2.1. NDH-1 Dehydrogenase

The NDH-1 dehydrogenase complex is a redox-driven proton pump linked with respiration and cyclic photosynthetic electron flow around PSI in cyanobacterial cells [279,280]. This complex reduces PQ and couples the released free energy to proton pumping across the membrane to drive active transport and synthesis of ATP [271,281]. Interestingly, the cyanobacterial NDH-1 uses reduced Fd and is a ferredoxin:plastoquinone oxidoreductase rather than a genuine NAD(P)H dehydrogenase (Figure 4) [281,282]. However, this complex may also use NAD(P)H, since it has been found to perform NADPH-dependent electron transfer to PQ [123]. The NDH-1 complex is present in heterocyst thylakoid membranes, being more abundant in heterocysts than in vegetative cells, which indicates that it may play an important role in heterocyst bioenergetics and metabolism [220,234,283,284].

Cyanobacterial NDH-1 seems to exist in two different forms involved in respiration and cyclic photosynthetic electron flow, which are termed NDH-1_1_ and NDH-1_2_ [280,281]. Both forms show a global architecture that closely resembles the typical L shape of the respiratory complex I [285,286,287,288], comprising a hydrophilic domain, which harbors three [4Fe–4S] clusters for electron transfer and a PQ binding site, that is connected to a wide membrane domain [280,281]. The hydrophilic domain of both NDH-1 forms comprises subunits NdhS, NdhHIJK, and NdhO and harbors the Fe–S clusters N6a, N6b, and N2 (Table 4) [271]. However, in contrast to the respiratory complex I, which receives electrons from NADH via FMN and a long electron chain of nine Fe–S centers placed in the sequence order N1a/N3 → N1b → N4-N7 → N5 → N6a → N6b → N2 [289], NDH-1 receives electrons from Fd and lacks both FMN and the six initial metal clusters [280,281,282]. Moreover, the NDH-1 complex operates with PQ [281], whereas the respiratory complex I requires ubiquinone [290] or menaquinone [291]. The membrane domain of the cyanobacterial NDH-1 complex is a proton-pumping machine and comprises NdhABCEG, NdhLMN, NdhPQ, and two additional subunits that vary in the isoform complexes NDH-1_1_ and NDH-1_2_ [280,281]. NDH-1_1_ harbors the subunits NdhD1 and NdhF1, whereas NDH-1_2_ exhibits the subunits NdhD2 and NdhF2. Subunits NdhB, NdhD, and NdhF are homologous to the antiporter-like subunits of the respiratory complex I and, therefore, are likely key subunits of the proton-pumping machinery in combination with the subunit NdhA [271]. NDH-1_1_ is the constitutive cyanobacterial isoform, while NDH-1_2_ is expressed in particular environmental conditions such as CO_2_ limitation or Fe depletion [292,293]. However, it is unknown whether both isoforms are expressed in heterocysts or one is preferentially used. Moreover, NDH-1_2_ may also withdraw the excess of electrons in the respiratory and cyclic photosynthetic electron chain by catalyzing a reverse electron flow using the proton-motive force and PQH_2_ to reduce NAD(P)^+^ or Fd [281]. 

NdhS is homologous to the Fd-docking domain found on the cytoplasmic side of PSI and mediates binding of Fd to NDH-1 and the delivery of electrons directly to the Fe–S cluster N6a in the subunit NdhI [280,281,294]. The three metal centers assemble an electron chain involved in electron transfer from Fd to the PQ pool that follows the transfer sequence Fd → N6a → N6b → N2 → PQ (Figure 4; Table 4) [280,289]. Clusters N6a and N6b are coordinated to four Cys residues each provided by the subunit NdhI, whereas the center N2 is coordinated to four Cys residues in the subunit NdhK. Interestingly, the cluster N2 is predicted to be pH-independent in the cyanobacterial NDH-1 complex [280], in contrast to the pH-dependence exhibited by the cluster N2 in the respiratory complex I [295]. The PQ-binding site is located in a cavity formed by subunits NdhIKH from the hydrophilic domain and the subunit NdhA from the membrane domain; they are in close proximity to the N2 Fe–S cluster at the NdhH/NdhK interface [280]. However, this cavity may contain a second PQ-binding site. Electrons are ultimately channeled to PQ molecules and specific residues of the cavity, in the form of a His and a Tyr, act as local proton donors for the reduction of PQ to PQH_2_, thus dissociating from the cavity and releasing to the membrane upon two-electron reduction and proton transfer [296,297]. The His residue also interacts with an Asp in the cavity forming a tight ion-pair, but PQH_2_ formation triggers the dissociation of such interaction, leading to energy release and conformational changes in a conserved network of nearby charged residues that could propagate across the NDH-1 membrane domain [280]. Thus, this energy release and the conformational changes may be employed by the NDH-1 complex to drive proton pumping across the membrane through the subunits NdhA, NdhB, NdhD, and NdhF.

NDH-1 complex provides an alternative supply of electrons to the PQ pool in the absence of PSII in heterocysts, functioning in close relationship with PSI [281], and contributes to build a membrane potential in the thylakoidal membranes that drives active transport and synthesis of ATP for N_2_ fixation and other metabolic processes in heterocysts (Figure 4 and Figure 3) [85,271]. Interestingly, heterocysts prefer the intracytoplasmic membranes as the site for the respiratory electron transport chain, while unicellular N_2_-fixing species preferentially use the cytoplasmic membrane [298,299]. Nevertheless, despite the fact that our understanding of the NDH-1 complex composition and functioning in cyanobacteria has greatly improved and revealed a high degree of complexity, its physiological function in heterocysts remains unclear. Thus, future work should clarify the roles of the respiratory electron transport chain and its adaptation to the specific metabolism and bioenergetics in heterocysts [281].

#### 4.2.2. Respiratory Terminal Oxidases

In cyanobacteria, respiratory terminal oxidases are Fe-containing enzymes that catalyze the four-electron reduction of O_2_ to H_2_O using electrons provided by either PQH_2_, PC, or cytochrome *c*_6_ [300,301,302]. They are grouped in three unrelated protein families classified as the heme-copper oxidase superfamily, the cytochrome *bd*-type quinol oxidase family, and the alternative oxidase family [146]. All heterocyst terminal oxidases contribute to the generation of ATP for the demanding diazotrophic metabolism and consume traces of O_2_ that enter the cells, despite the thick heterocyst envelope.

Heme-copper oxidases are redox-driven proton pumps that couple the four-electron reduction of O_2_ into H_2_O to the translocation of protons across membranes [145]. This electrochemical gradient is then exploited by ATP synthase to generate a readily available energy source for cellular processes in heterocysts (Figure 4). Heme-copper oxidases can use cytochromes [301,302], type-1 (or blue-) copper proteins [301,303,304], or quinols [300] as electron donors. They are classified as *aa*_3_-type cytochrome *c* oxidases (Cox), which are encoded by *coxBAC* and accept electrons from cytochrome *c* and copper proteins, and *bo*_3_-type quinol oxidases (Qox), which are encoded by *qoxBAC*, accept electrons from PQH_2_ and include the so-called alternative respiratory terminal oxidases (ARTO) [145,305,306,307]. Remarkably, heme-copper oxidases play a key role in the aerobic respiratory electron transport chain in heterocysts (Table 4).

Cyanobacterial *aa*_3_-type oxidases are three-subunit enzymes, but only subunits I (CoxA) and II (CoxB) harbor the metal-containing redox cofactors and contain the amino acid residues involved in O_2_ reduction and proton pumping. Thus, subunit III (CoxC) is likely not involved in catalysis [146,302]. CoxA contains 12 predicted transmembrane α-helices that harbor an O_2_-reduction central heme-Cu binuclear catalytic center, which contains a high-spin heme *a*_3_ electronically linked to a Cu atom (Cu_B_) and an additional low-spin heme *a* that interacts with heme *a*_3_ (Figure 4; Table 4) [145,302,308]. CoxA is involved in electron transfer through the metal-containing centers, proton pumping through Asp and Lys channels, and catalysis of the O_2_ reduction reaction [145]. The amino acid residues coordinating the metal centers are conserved in all cyanobacterial CoxA [145]. Thus, the heme-*a*_3_ Fe atom and Cu_B_ in the binuclear reaction center are primarily coordinated to four His residues and an additional Asp interacts with heme *a*_3_, while the Fe atom in heme *a* is coordinated to two His residues and the heme group further interacts with an Arg and a Tyr. The cyanobacterial CoxB exhibits two transmembrane α-helices and a soluble thylakoid lumen domain and contains a Cu center (Cu_A_) with two mixed-valence, electronically-coupled Cu atoms (Figure 4; Table 4) [145,302,308]. CoxB is the docking site for soluble electron donors and also participates in both electron transfer and proton pumping [145]. The Cu–Cu center Cu_A_ is coordinated to two His, two Cys, one Met, and one Glu, where the two Cys residues bridge both Cu atoms (Figure 2G) [145]. Interestingly, the amino acid Glu is coordinated to one of the Cu atoms through the carbonyl group of the peptide bond [145]. Cu_A_ is the main docking site for the soluble electron donors PC and cytochrome *c*_6_ and it is involved in the primary electron transfer to the cytochrome *c* oxidase complex (Figure 4) [309,310,311,312]. Electrons are then transferred from Cu_A_ in CoxB to heme *a* and consecutively to the heme *a*_3_–Cu_B_ center of CoxA, which is the catalytic site of cytochrome *c* oxidases where O_2_ is ultimately reduced to H_2_O [310].

Qox (or ARTO) complexes are also members of the heme-copper superfamily, as the residues responsible for binding and complexation of the binuclear heme-Cu center are conserved. However, on the basis of subunit composition, they are more similar to cytochrome *bo*_3_-quinol oxidases, as the Qox subunit II homologue lacks the Cu_A_ site, despite being highly conserved where this site would localize [306]. Cyanobacterial *bo*_3_-type quinol oxidases are considered three-subunit enzymes, although it is not clear whether they may contain more subunits [128,143,145]. They show high similarities to *aa*_3_-type cytochrome *c* oxidases, but only subunit I (QoxA) exhibits metal-containing redox cofactors, while subunits II (QoxB) and III (QoxC) play structural roles [145]. As in CoxA subunits from *aa*_3_-type oxidases, cyanobacterial QoxA harbors an O_2_-reduction, heme-Cu central binuclear catalytic site containing a Cu_B_, which is electronically coupled instead to a high-spin type-*o*_3_ heme, and an additional low-spin type-*b* heme, rather than a heme *a*, that interacts with heme *o*_3_ (Figure 4; Table 4) [300,307]. Moreover, QoxA also exhibits the amino acid residues involved in O_2_ reduction and proton pumping as in CoxA subunits [145]. In contrast to CoxB from *aa*_3_-type oxidases, QoxB has neither a binuclear Cu_A_ center nor a binding site for soluble electron carriers such as PC and cytochrome *c* [300,311]. The location of the Cu_A_ center found in CoxB is blocked in QoxB by hydrophobic residues, thus preventing access from the thylakoid luminal side. Instead, heme *b* receives electrons directly from a membrane PQH_2_ molecule that are then sent to the heme *o*_3_-Cu_B_ catalytic center of quinol oxidases, where O_2_ is ultimately reduced to H_2_O [145,313]. Protons generated upon PQH_2_ oxidation are released on the thylakoid lumen contributing to the membrane electrochemical gradient (Figure 4). Amino acid residues coordinating the metal cofactors heme *o*_3_, Cu_B_, and heme *b* are conserved in QoxA subunits, being the heme-*o*_3_ Fe atom and Cu_B_ in the binuclear reaction center coordinated to four His residues and the heme-*b* Fe atom coordinated to two His residues [145,300]. 

Three heme-copper oxidases, namely two *aa*_3_-type cytochrome *c* oxidases (Cox1 and Cox2) and one *bo*_3_-type oxidase (Qox, also called ARTO and formerly known as Cox3), are found in *Anabaena* sp. PCC 7120. While Cox1 is expressed only in vegetative cells, Cox2 and Qox are expressed exclusively in heterocysts and are essential for diazotrophic growth (Table 4) [127,128,146]. Cox2 is encoded by the operon *coxB2-coxA2-coxC2* and has recently been considered a type-2 ARTO, although they catalyze the same cytochrome *c*-O_2_ oxidoreductase reaction as mitochondrial-type cytochrome *c* oxidases [146]. In turn, Qox is a type-1 ARTO encoded by the operon *alr2729-alr2730-coxB3-coxA3-coxC3* [127,128,145,146,305]. Both Cox2 and Qox complexes are located in the honeycomb membranes of heterocysts and contribute to respiration to generate ATP, protect the N_2_-fixation machinery against O_2_, and are required for the normal development and performance of heterocysts [128,145,146].

*Anabaena* sp. PCC 7120 further contains one *bd*-type quinol oxidase encoded by the *all4023*-*all4024* (*cydBA*) gene cluster [146,314]. Tri-heme cytochrome *bd* oxidases are complexes made of two integral membrane subunits (CydAB) and contain two *b*-type hemes, namely *b*_558_ and *b*_595_, and one heme *d*, but lack Cu atoms or non-heme Fe (Figure 4; Table 4) [315,316]. They couple PQH_2_ oxidation to four-electron reduction of O_2_ to H_2_O in a reaction that generates a proton-motive force via transmembrane charge separation rather than direct proton pumping across membranes, as opposed to heme-copper oxidases [317,318,319,320]. Subunit I (CydA) contains nine predicted transmembrane α-helices, binds all three redox metal centers, and is involved in electron transfer from PQH_2_ to O_2_ through such metal clusters [315,319]. Subunit II (CydB) exhibits eight predicted transmembrane α-helices and is mainly involved in structural roles [315,319]. CydA harbors a di-heme active site composed of the high-spin pentacoordinate hemes *b*_595_ and *d*, which are electronically coupled, and the low-spin hexacoordinate heme *b*_558_, which transfers electrons from the electron donor PQH_2_ towards the di-heme active site (Figure 4; Table 4) [315,319]. CydA exhibits a soluble domain on the *P*-side of the membrane that is termed Q loop and is involved in PQH_2_ oxidation and electron transfer to heme *b*_558_. In the active site, heme *b*_595_ receives electrons from heme *b*_558_, while heme *d* is responsible for O_2_ binding and, in partnership with heme *b*_595_, catalyzes the O_2_ reduction reaction [315]. O_2_ reduction into H_2_O also requires a proton channel that seems to be formed by some amino acid residues in CydA. Although CydB appears to have mainly structural functions, its N-terminal portion could also interact with hemes *d* and *b*_595_ and the active site of cytochrome *bd* oxidases could be located at the subunit interface [321]. However, the amino acid residues involved in the coordination of the three metal centers are provided exclusively by CydA and are conserved in the entire family of cytochrome *bd* oxidases [315,319]. Therefore, the axial ligands of the hexacoordinate heme-*b*_558_ Fe atom are a His and a Met, whereas pentacoordinate Fe atoms in hemes *b*_595_ and *d* are coordinated to a single His or Glu, respectively.

Cytochrome *bd* oxidases exhibit a very high affinity for O_2_ [319], although they are half as efficient in terms of energy conservation in comparison to heme-copper quinol oxidases [322]. Moreover, this oxidase is localized both in the cytoplasmic and thylakoidal membranes in cyanobacteria [268,315], is expressed at higher levels under microaerobic conditions in *Anabaena* sp. PCC 7120 [323], and is also essential for diazotrophic growth in heterocyst-forming cyanobacteria, playing different roles in heterocysts [146,314]. Cytochrome *bd* oxidase seems to prevent overreduction of the PQ pool and is important to regulate the overall electron transport chains, which could otherwise lead to O_2_ radical formation and damage of PSI, electron transport complexes, and other proteins in heterocysts (see Section 5) [146]. Moreover, it is also an O_2_ scavenger to avoid the inactivation and degradation of O_2_-sensitive enzymes such as nitrogenases and hydrogenases in heterocysts [146,314,315]. 

The alternative oxidase family includes plastid- and mitochondrial-type quinol terminal oxidases [146]. Plastid-type oxidases, also known as plastoquinol terminal oxidases (PTOX), are single-subunit membrane metalloproteins attached to thylakoid membranes on the cytoplasmic side [268,278,324]. They couple the oxidation of PQH_2_ to the four-electron reduction of O_2_ to H_2_O and display a structurally conserved four-α-helix bundle that harbors a catalytic non-heme Fe–Fe center (Table 4) [324,325,326]. PTOXs are anchored to the membrane by two additional amphipathic α-helices that are buried in the membrane and exhibit a Tyr residue which has been suggested to be involved in PQH_2_ binding [327,328]. Moreover, the two Fe atoms of the active site are coordinated to six conserved residues including two His and four Glu [324,326,328]. PTOX functions as a scavenger for O_2_ to prevent over-reduction of the PQ pool and formation of reactive oxygen species [328] and because the respiratory and photosynthetic electron transport chains share components in cyanobacteria, PTOX could play an important role in the regulation of the electron flux through both transport chains as an electron safety valve [328]. Thus, PTOX could be involved in an alternative electron transfer route that mediates electron flow from PQH_2_ to O_2_ [328,329].

In heterocyst-forming cyanobacteria such as *Anabaena* sp. PCC 7120 and *Anabaena variabilis* ATCC 29413, *ptox* gene homologues are found [328]. The information available about PTOXs in cyanobacteria and heterocysts is scarce, but it has been suggested that PTOX might be expressed in heterocysts in *Anabaena* sp. PCC 7120 [329]. PTOX serves as an alternative terminal oxidase [319] and might contribute to maintain an O_2_-free environment in heterocysts in cooperation with the flavodiiron protein Flv3B (see next section) [257], thus enabling N_2_ fixation to function efficiently [329]. However, the localization of PTOX in heterocysts has yet to be confirmed.

### 4.3. Flavodiiron Proteins

The contribution of Fe to photosynthesis is also manifested by flavodiiron proteins (FDPs) [330,331,332], formerly known as A-type flavoproteins (Flv) [333]. They are a large family of modular enzymes that display sequence similarity and are present in anaerobic and some aerobic archaea and bacteria, but also in protozoans and some photosynthetic eukaryotes [330,334,335]. FDPs share an N-terminal metallo-β-lactamase domain followed by a flavodoxin domain, which represent the common core, and are classified based on the additional C-terminal domains as classes A, B, C, and D [336]. The majority of FDPs belong to class A, which groups the prototype enzymes and the simplest forms with the shortest extension sequences, representing the minimal core structure, and are found in bacteria, archaea, and protozoans. Class-B FDPs are found in enterobacteria, whereas class-D FDPs are present in some bacteria and protozoans. Class-C FDPs appear to be specific to cyanobacteria, although some eukaryotic oxygenic phototrophs also contain members of this class [336,337,338].

Cyanobacterial FDPs operate as cytosolic hetero- and homodimers and are involved in the regulation of the photosynthetic electron transport chain through photoprotection of photosystems [276,330]. They are present in all cyanobacteria, but some unicellular species only contain two *flv* genes such as *flv1a* and *flv3a*, while filamentous heterocyst-forming cyanobacteria exhibit four to six *flv* genes, two of them expressed exclusively in heterocysts, namely *flv1b* and *flv3b*, being Flv3B specially relevant for diazotrophic growth (Table 5) [257]. Cyanobacterial members exhibit three conserved structural portions, namely an N-terminal metal-containing β-lactamase domain, which harbors a non-heme Fe–Fe center where O_2_ reduction is catalyzed, a central flavodoxin domain containing a flavin mononucleotide moiety, and an additional C-terminal NAD(P)H:flavin reductase domain that enables cyanobacterial FDPs to directly use NAD(P)H as an electron donor to reduce O_2_ [336,338].

FDP structures display a head-to-tail arrangement where the Fe–Fe site of one monomer and the FMN of the other monomer closely contact each other, ensuring a fast electron transfer between the two redox centers [338,339,340,341]. About half of cyanobacterial FDPs are grouped as class-C, type-1 FDPs and display a canonical coordination of the Fe–Fe center, which is similar to that of class-A members, via ligands of conserved amino acid residues such as four His, two Asp, and one Glu [338,341,342]. The heterocyst-specific Flv3B protein belongs to this group and exhibits an Fe–Fe center, where both Fe atoms are held in close proximity by an Asp residue and each Fe atom is coordinated individually to one Glu or Asp and two His residues (Figure 2H) [330,338,341,342]. Moreover, both Fe atoms are bridged by a μ-oxo or μ-hydroxo species in the oxidized diferric center [338,342]. Other cyanobacterial members display non-canonical metal-binding motifs with significant variations in such residues and are grouped in 11 additional class-C types [338,342]. The heterocyst-specific Flv1B protein belongs to the class-C, type-3 group and contains an Fe–Fe center that could be coordinated to Ser, Asn, Lys, two Arg, and two His residues [330,338,343]. However, little is known about the involvement of these multiple putative ligand substitutions in the coordination of Fe–Fe centers and further research is required to decipher their function.

As with Flv1A and Flv3A FDPs from vegetative cells, heterocyst-specific FDPs, Flv1B and Flv3B, seem to function as NAD(P)H oxidoreductases that perform a light-dependent, four-electron transfer reaction and convert O_2_ into H_2_O without the formation of reactive oxygen species [276,330,331,341]. However, the electron donor of heterocyst-specific FDPs might also be Fd (Table 5) [257]. This reaction would ensure a proper microaerobic environment in heterocysts for light-induced protection of the N_2_-fixation apparatus in the cytoplasm of heterocysts. Moreover, in vegetative cells, Flv1A and Flv3A form a heterodimer that could also sequester electrons from the acceptor side of PSI and utilize them to reduce O_2_, acting as a strong electron sink [331,344]. This mechanism could also take place in heterocysts, where heterocyst-specific FDPs could safeguard PSI from photodamage under fluctuating light conditions to prevent its over-reduction, regulate the flux of electrons through the photosynthetic apparatus, and maintain the redox balance of the electron transport chains [257,330]. Nevertheless, the heterocyst-specific Flv3B seems to operate as a homodimer and Flv1B does not seem to have a clear role in heterocysts, thus further research is required to elucidate their specific function. The heterocyst-specific FDP Flv3B may participate in the control of the redox status of the cytosol by removing O_2_ and provide the appropriate conditions for the function of the nitrogenase, and probably many other enzymes, under illumination [257].

The role of FDPs transferring light-driven electrons from PSI directly to O_2_ can be important in heterocysts to prevent oxidative damage of the N_2_-fixing machinery and to create the appropriate redox conditions for heterocyst metabolism [257]. Light-induced O_2_ uptake performed by FDPs in heterocysts is essential for optimal nitrogenase performance and, therefore, for an appropriate supply of nitrogen in the form of amino acids in the diazotrophic filament. Thus, when heterocysts cannot provide the vegetative cells with nitrogen compounds, nitrogen starvation in the diazotrophic filament may cause an accumulation of succinate, fumarate, and malate in the Krebs cycle and an increase in respiratory activity to dissipate the excess of carbon [257]. Terminal respiratory oxidases located in the honeycomb membranes and cytoplasmic FDPs consume O_2_, contributing to create a micro-oxic environment in heterocysts [127,257]. However, scavenging of O_2_ by FDPs and terminal respiratory oxidases seems not to be redundant [257]. Thus, whereas the terminal respiratory oxidases likely remove O_2_ in the heterocyst poles, since the respiratory activity is located at the honeycomb membranes [115], heterocyst FDPs are cytoplasmic [345].

Heterocysts contain only the small isoform of FNR, which lacks the phycobilisome-binding domain and is active in oxidation of NADPH, and do not seem to reduce NADP+ on the donor side of PSI [257,346]. Moreover, it is considered that both NADH and NADPH are produced by glycolysis and the OPPP, respectively, equally in darkness and under the light [257]. In addition to both electron donors, ferredoxin FdxH delivers electrons to nitrogenase and has been suggested to play a role as a common pool where reducing equivalents from different pathways are targeted to [96]. This Fd is then further tunneled to N_2_ fixation [96], but could also act as an electron donor for Flv3B [257]. Thus, it has been proposed that illumination modifies the redox status of heterocysts and activates the Flv3B-mediated electron transfer from the reducing side of PSI, likely from FdxH, to O_2_. In this case, Flv3B would use light-driven electrons originating from reductants provided by vegetative cells [257].

## 5. Metalloproteins in Oxidative Stress Management

Reactive oxygen species (ROS) are byproducts of aerobic metabolism and photosynthesis. The respiratory machinery generates ROS via auto-oxidation of flavin cofactors, while the photosynthetic electron transport chain does so through the photosystem complexes [46,347,348]. ROS are unavoidably generated by accident as intermediates of O_2_ reduction, including the superoxide anion (O_2_^•^^−^), hydrogen peroxide (H_2_O_2_), and the hydroxyl radical (OH^•^), or by energization of O_2_ from photosensitized chlorophylls, including singlet oxygen (^1^O_2_); all of them are powerful oxidizing agents [46,347]. However, they have different intrinsic features, exhibiting distinct reactivities, toxicity levels, and targets. O_2_^•−^ radicals can damage Fe–S clusters in metalloproteins, releasing Fe^2+^ and H_2_O_2_ in the process and, thus, an efficient O_2_^•−^ detoxification system in the form of superoxide dismutase is essential to catalyze the disproportionation of O_2_^•−^ into O_2_ and H_2_O_2_ [46,348,349]. Although this reaction represents the major source of intracellular H_2_O_2_, this ROS is also produced by multiple oxidases that use O_2_ for redox reactions [131]. H_2_O_2_ can damage Cys and Met residues and Fe–S clusters and also react with Fe^2+^ to form Fe^3+^ and OH^•^ through the Fenton reaction. OH^•^ radicals are very reactive and lethal for cells, thus, the detoxification of H_2_O_2_ through peroxidases and/or catalases, which convert it into H_2_O and O_2_, is crucial [348,350]. Moreover, Fe^2+^ can be regenerated (and can react again with H_2_O_2_, producing more OH^•^) through a reaction between Fe^3+^ and O_2_^•−^, which is known as the Haber–Weiss reaction in combination with the Fenton reaction [131,351], but also through a reaction between Fe^3+^ and H_2_O_2_ that produces Fe^2+^ and hydroperoxyl radicals (HOO^•^), which are the protonated forms of O_2_^•^^−^ radicals [352]. All of these reactions involving Fe and ROS are known as Fenton chemistry reactions. If not scavenged effectively, ROS can damage nucleic acids, proteins, membrane lipids, and other cellular components and affect the overall cellular homeostasis in cyanobacteria by inactivating the protein synthesis machinery and altering thylakoid and plasma membranes permeability via lipid peroxidation of unsaturated fatty acids [131,353].

Despite the morphological and metabolic adaptations of heterocysts to limit their intracellular O_2_ content, traces of this gas still permeate through the envelopes and/or from neighbor vegetative cells [131,354,355]. Therefore, ROS are also inevitably generated in heterocysts by both the respiratory and the PSI-dependent photosynthetic electron transport chains, being especially relevant in the latter when the intensity of light-driven electron transport outpaces the rate of electron consumption by nitrogenase and other metabolic pathways [46,131,356]. Thus, heterocysts produce O_2_^•−^ through electron leakage from flavoproteins to O_2_ and at the reducing side of PSI via one-electron reduction of O_2_ [46,348,349,355]. The nitrogenase reductase can also generate H_2_O_2_ under micro-oxic conditions through an auto-protection mechanism in order to prevent its inhibition by oxidative damage [356,357]. Thus, when the molar ratio of NifH and O_2_ is greater than 4:1, the nitrogenase reductase can reduce O_2_ to H_2_O_2_ without being inactivated by O_2_ [357]. The H_2_O_2_ generated through this reaction needs to be removed subsequently by H_2_O_2_-scavenging enzymes [357]. These conditions seem to be met within heterocysts, since the concentration of NifH is estimated to be much greater than four times the O_2_ concentration [61,76]. Therefore, despite the fact that the nitrogenase reductase can uptake O_2_, thus preventing partially its inactivation and the generation of ROS, this enzyme can also contribute to H_2_O_2_ formation [61,356,358]. Since heterocysts have a very active respiratory metabolism, exhibit a high concentration of nitrogenase reductase, and are exposed to changing external conditions such as light intensity fluctuations, their ability to perceive ROS and to rapidly initiate antioxidant defenses is crucial to avoid oxidative damage that otherwise would impair their adequate functioning and the survival of the diazotrophic filament [46,76,128]. Therefore, heterocysts express various specific metalloproteins to scavenge ROS, such as superoxide dismutases, peroxidases, and Dps (DNA-binding protein from starved cells) proteins, showing an elaborate enzymatic machinery to defend the cellular components from ROS (Table 6). However, heterocysts do not seem to contain catalases, unlike vegetative cells in the diazotrophic filament [131,359,360]. The physiological role of such ROS-detoxifying enzymes is of particular interest in heterocysts due to the high sensitivity of the nitrogenase enzyme to both O_2_ and ROS [354,361,362].

### 5.1. Superoxide Dismutases

Superoxide dismutases (SODs) are the first line of defense to alleviate oxidative stress produced by O_2_^•^^−^ in aerobic organisms [363]. They are classified according to their metal cofactor as Mn– (SodA), Fe– (SodB), Cu–Zn– (SodC) and Ni–SOD (SodN) [46,364,365,366,367]. Moreover, cambialistic SODs that can use either Fe or Mn in their active sites have been found [350,368]. Fe– and Mn–SODs display a very similar structure, which explains why some Fe–SODs can function with a Mn atom replacing Fe [369]. All SOD forms are present in cyanobacteria with the exception of cambialistic enzymes, which have not been found in this group of microorganisms to date [350]. Some unicellular species house only Fe–SOD, whereas genes encoding Ni–SOD, and very rarely Cu–Zn–SOD, are mostly present in marine strains [350,368]. Interestingly, most unicellular non-N_2_-fixing strains seem to possess a single SOD form [368], while N_2_-fixing heterocystous species such as *Anabaena* sp. PCC 7120, *Nostoc punctiforme* ATCC 29133, and *Anabaena variabilis* ATCC 29413 have a combination of Fe–SOD and Mn-SOD to scavenge O_2_^•^^−^ [354,365,368,370,371,372], which could provide a nitrogen-status-specific protection in diazotrophic filaments [131,371].

The canonical forms of Mn–SOD and Fe–SOD are structurally distinct from Cu–Zn–SOD and Ni–SOD. Both Mn– and Fe–SOD are typically homodimers and share a common topology and identical metal-chelating residues at the active site with a high degree of sequence and structural homology, except for slight differences in amino acid residues [131,368]. Cyanobacterial Mn– and Fe–SODs share a similar mononuclear active site with one atom of Mn or Fe, respectively, which is coordinated in both cases to three His, one Asp, and a fifth ligand provided by a solvent molecule that can be H_2_O or OH^−^, depending on the oxidative state of the metal cofactor (Figure 2I for Mn–SOD metal center) [368]. Moreover, one Glu and one Tyr generally form a dimer surface spanning the interface and bridging the active metal sites between the opposite halves of each subunit [368]. Mn– and Fe–SODs convert the highly toxic and reactive O_2_^•−^ radicals into a more stable H_2_O_2_ via a dismutation reaction [46]:

2O_2_^•−^ + 2H^+^ → H_2_O_2_ + O_2_.

Heterocysts harbor both a Mn–SOD encoded by *sodA* and an Fe–SOD encoded by *sodB* (Table 6) [220,350,354,365]. Both metalloenzymes differ in their cofactor requirement and their localization. Mn–SOD contains an N-terminal signal peptide and is localized in the thylakoid and cytoplasmic membranes [131,372]. Moreover, it is also cleaved from those membranes under N_2_-fixing conditions and the soluble Mn–SOD is distributed in the cytosol, the thylakoid lumen, and the periplasm, both in heterocysts and vegetative cells [373,374]. Fe–SOD is also present in both cell types in diazotrophic filaments but, in contrast to Mn–SOD, is only located in the cytosol [354,365,370]. While all SODs are soluble enzymes, the cyanobacterial Mn–SOD is the only member to be membrane-anchored by a transmembrane helix [350,354,365,372,375,376]. On the other hand, although the periplasmic and luminal Mn–SODs form homodimers, the soluble cytosolic Mn– and Fe–SOD enzymes form active homodimers and heterodimers [354,365,370,374].

The high sensitivity of nitrogenase to O_2_^•−^ radicals [361] requires the presence of active SODs in heterocysts to avoid nitrogenase oxidative damage [377]. Therefore, an active Mn–SOD in the thylakoidal membranes and lumen, as well as in the periplasm and the plasma membrane, enables the sequestration of O_2_^•−^ radicals generated due to PSI activity and the respiratory electron chains at the site itself, minimizing any leakage to the cytoplasm where it could target the nitrogenase, but also other proteins, nucleic acids, and other biomolecules [354,355,365,371,374]. Moreover, the cytosolic form of Mn–SOD and the cytosolic Fe–SOD contribute to the elimination of any O_2_^•−^ traces in the cytoplasm. Therefore, while Fe–SOD has a major role in the degradation of O_2_^•−^ radicals in the cytosol, Mn–SOD scavenges O_2_^•−^ in the three different heterocyst compartments: Periplasmic space, cytosol, and thylakoid lumen [131,374]. Thus, the cooperative functioning of individual SODs in specific cellular compartments contributes to maintain an appropriate redox status in heterocysts. In turn, the degradation of H_2_O_2_ in heterocysts is performed by rubrerythrin and Dps proteins.

### 5.2. Peroxidases

Although H_2_O_2_ is the most stable ROS, it still exhibits a high reactivity. Thus, a wide variety of scavenging enzymes have evolved to remove such a ROS [378]. Peroxidases are a large family of oxidoreductases involved in the reduction of H_2_O_2_ and organic peroxides and are classified as heme and non-heme peroxidases [47]. Heme-containing peroxidases are further divided as monofunctional catalases [379], heme peroxidases [380,381], peroxidase-cyclooxygenases [382], and other minor groups of heme-containing peroxidases, including the novel dye-decolorizing peroxidase family [383] or bacterial di-heme peroxidases [384]. Non-heme peroxidases include Mn catalases [379], V peroxidases [385], erythrins, rubrerythrins, and Dps proteins [386], and ubiquitous thiol peroxidases, including peroxiredoxins and glutathione peroxidases, which catalyze the reduction of peroxides by catalytic Cys residues and thiol-containing proteins as reductants [387].

Heterocysts contain specific metalloenzymes with peroxidase activity, such as a rubrerythrin and multiple Dps proteins, but do not seem to house catalases, despite the fact that heterocyst-forming cyanobacteria contain two genes (*katA* and *katB*) encoding binuclear Mn–catalases [47,131,359,360]. Heterocysts also harbor multiple peroxiredoxins that represent an important line of their defense against ROS [131]. However, these enzymes are thiol peroxidases rather than metalloenzymes and, therefore, are not included in this review. While catalases reduce H_2_O_2_ producing H_2_O and O_2_, enzymes with peroxidase activity catalyze H_2_O_2_ reduction generating only H_2_O. Thus, in the context of the reduced O_2_ cellular environment required in heterocysts for the enzyme nitrogenase, the absence of catalases may confer a distinct advantage [359,360]. Hence, enzymes that detoxify H_2_O_2_ without generating O_2_ could be better suited to function in heterocysts than catalases. It is worth mentioning that, while catalase activity was found in heterocysts of *Anabaena cylindrica* [389] and *Nostoc muscorum* [390], subsequent studies could not confirm the expression of catalase genes in heterocysts [131,359,360], at least in heterocysts of *Anabaena* sp. PCC 7120. However, to the best of our knowledge, the expression of *katA* (*alr0998*) has not been analyzed in diazotrophic conditions in *Anabaena* sp. PCC 7120 and, therefore, it remains unclear whether this gene is expressed in heterocysts [131,359].

#### 5.2.1. Rubrerythrin

Rubrerythrins are non-heme tri-Fe enzymes involved in oxidative stress protection as H_2_O_2_ scavengers in cyanobacteria, but are predominantly found in anaerobic organisms rather than aerobic species [349,356,391,392]. They consist of an N-terminal erythrin domain with a four-α-helix bundle configured in two anti-parallel-helix pairs, which belongs to the ferritin-like superfamily, and a small C-terminal rubredoxin domain [386]. The erythrin domain contains a permanent catalytic non-heme Fe–Fe center within the bundle that is involved in the reaction to detoxify H_2_O_2_, while the rubredoxin domain harbors a mononuclear [Fe–4S] cluster that delivers electrons to the reaction metal center [35,386,393,394]. The Fe–Fe center is coordinated to seven highly conserved residues within the four-α-helix structure in erythrin members, involving two His and four Glu residues in the reduced diferrous center (Figure 2J, left), or one His and five Glu residues as ligands for the oxidized diferric site (Figure 2J, right) [349,356,386,393,394]. This redox-dependent Glu–His ligand switching at the Fe–Fe center is unique among di-iron proteins. Interestingly, both atoms of Fe are bound to two H_2_O molecules in the reduced diferrous site or linked by a μ-oxo bridge in the oxidized metal center (Figure 2J) [349,393,394]. In turn, the single Fe atom in the rubredoxin domain is coordinated to four Cys residues forming its characteristic mononuclear Fe–Cys_4_ center (Figure 2L) [386,393,395].

Rubrerythrins form homodimers that could be involved in different chemical mechanisms of protection against oxidative stress [349,356,396], although the most broadly observed role is as a catalyst in the reduction of H_2_O_2_ into H_2_O via electrons transferred from NAD(P)H. In the reaction cycle of rubrerythrin to reduce H_2_O_2_, the catalytic Fe–Fe center is reduced via two consecutive intramolecular one-electron transfers from the rubredoxin [Fe–4S] cluster, which in turn is reduced by NAD(P)H, but a concerted electron transfer process to the catalytic site favors the two-electron reduction reaction of H_2_O_2_ over a one-electron, Fenton-type redox reaction [349]:

H_2_O_2_ + 2*e*^−^ + 2H^+^ → 2H_2_O.

Heterocysts harbor a specific rubrerythrin enzyme (RbrA, Alr1174 in *Anabaena* sp. PCC 7120; Table 6) that is poorly expressed in vegetative cells in the diazotrophic filament [356]. RbrA exhibits only a moderate similarity to rubrerythrins from anaerobic bacteria, but the amino acid residues required to coordinate the catalytic Fe–Fe center and the mononuclear Fe are conserved [356]. This enzyme has a strong peroxidase activity using electrons driven from NADPH, indicating that it has similar biochemical functions to those of anaerobic organisms [356]. However, unlike rubrerythrin members from anaerobic bacteria, RbrA requires the participation of the enzyme FNR for electron transfer from NADPH. Thus, FNR functions as a RbrA:NADPH oxidoreductase and interact with the erythrin domain reducing the Fe–Fe center without the participation of the [Fe–4S] cluster of the rubredoxin domain [356]. RbrA functions in H_2_O_2_ decomposition within heterocysts and is essential for the protection of the nitrogenase from H_2_O_2_ damage and the optimal growth of the diazotrophic filament.

#### 5.2.2. Dps Proteins

Dps proteins are part of the ferritin-like superfamily, which includes proteins with a wide variety of roles such as iron storage and/or detoxification in members of the ferritin family (ferritins, bacterioferritins, and Dps proteins), ROS scavenging in enzymes of the erythrin/rubrerythrin and Mn–catalase families, and ubiquinone biosynthesis in proteins of the COQ7 family, all of them sharing the same distinctive structural motif [386].

The subunit structure of Dps proteins displays the ferritin-like, four-α-helix bundle core present in rubrerythrins, although a fifth helix sits in a loop connecting the two anti-parallel-helix pairs perpendicularly to the four-α-helix bundle [386]. Dps complexes comprise 12 subunits that form a large, quasi-spherical, shell protein that defines an internal cavity of ~4.5 nm in diameter, and structurally resemble ferritins and bacterioferritins, although the latter two are assembled from 24 identical or highly similar subunits [386,397,398]. Dps proteins contain unique inter-subunit catalytic Fe–Fe centers that are located at the interface between two-fold symmetry-related subunits in the dodecamer complex, rather than within the four-α-helix bundle of each subunit as for other members of the ferritin-like superfamily, such as rubrerythrins, bacterioferritins, and ferritins [399]. Each subunit dimer harbors two identical Fe–Fe center sites, with the Fe atoms of each site coordinated directly to highly conserved residues within the Dps proteins, namely a His, Asp, and Glu provided by both dimer subunits, and two additional molecules of H_2_O which, in turn, interact with a conserved His and Glu pair (Figure 2K) [386,397,398,400]. Moreover, a μ-oxo bridge connects both Fe atoms in the ferric oxidation state, a feature found in functional di-iron proteins [386,397,398,400,401]. However, the two metal sites exhibit different affinity for Fe in Dps proteins. Thus, one metal site employs the His, Asp, and Glu as Fe-coordinating ligands and binds Fe tightly, whereas the other metal site binds Fe loosely via the same Glu residue and two H_2_O molecules that are further stabilized by the His and Glu pair (Figure 2K) [397,398,400,402].

Fe–Fe active sites in Dps proteins are also termed ferroxidase centers and are non-permanent Fe centers involved in the oxidation of free Fe^2+^ from the cytoplasm to disproportionate H_2_O_2_. Dps proteins function as peroxidase metallocomplexes catalyzing the reduction of H_2_O_2_ into H_2_O, while oxidizing Fe^2+^ into insoluble Fe^3+^ to prevent the harmful Fenton chemistry, which generates OH^•^ radicals via the reaction between H_2_O_2_ and Fe^2+^ [386,388,397,403]. H_2_O_2_ binds directly to Fe atoms at the ferroxidase center, generating an intermediate μ-oxo-bridged Fe-Fe species and, after oxidation, the ferric ion generated either migrates into the storage cavity or forms part of a catalytic center for further ferroxidation activity [386]. Therefore, Dps proteins are relevant as H_2_O_2_ scavengers but also as redox-stress protectors hampering the generation of dangerous radicals. Furthermore, assembled shells are pierced by channels at the subunit symmetry sites and through individual subunits, enabling the Fe^3+^ ions derived from the reaction catalyzed at the ferroxidase centers to enter and exit the complexes [386,397,398]. The hollow center provides a cage to capture and store up to 500 atoms of Fe as a ferrihydrite-like mineral that can also incorporate phosphate [398,404,405]. Consequently, Dps protein shells also play a relevant role in Fe storage to control Fe homeostasis in the cell, while storing the Fe core in a soluble state that maintains this metal biologically available for immediate use in metalloproteins and also prevents nutritional deprivation and stress in cells [386,406]. Many Dps proteins also bind DNA non-specifically through the subunit terminal regions [397,407,408]. DNA is the major target for radicals induced by Fenton chemistry since its negative charge attracts Fe ions readily. Thus, Dps proteins represent a DNA protection mechanism that enables an efficient removal of Fe^2+^ and H_2_O_2_ to prevent the generation of ROS radicals and the subsequent oxidative damage in situ [386]. All features exhibited by Dps proteins greatly extend their role in ROS management and convert these metalloenzymes in more than simple peroxidases.

The heterocyst-forming cyanobacterium *N. punctiforme* ATCC 29133 contains four genes encoding Dps proteins that are expressed in heterocysts (Table 6) [220,409,410]. These proteins are involved in H_2_O_2_ scavenging, oxidative stress management and/or DNA binding and are termed NpDps1 (Npun_R3258), NpDps2 (Npun_F3730), NpDps3 (Npun_R5701), and NpDps4 (Npun_R5799). The corresponding proteins in *Anabaena* sp. PCC 7120 are All0458, All4145, All1173, and Alr3808, respectively [409]. NpDps1 is expressed in heterocysts at higher levels than in vegetative cells, but expression analyses of All0458 in heterocysts have not been performed [220,410,411]. However, the latter forms dodecamers and exhibits ferroxidase activity [411]. NpDps2 and All4145 are expressed in heterocysts, although the expression is weaker than in vegetative cells for both proteins [409,412]. NpDps2 is the major protein providing H_2_O_2_ tolerance to cells in *N. punctiforme* [409]. NpDps3 is expressed in heterocysts at higher levels than in vegetative cells but All1173 expression analyses in heterocysts have not been carried out [409,411]. However, All1173 forms 12-subunit complexes and exhibits ferroxidase activity, stores Fe, and protects DNA [409,411,413]. NpDps4 and Alr3808 are expressed in heterocysts at higher levels than in vegetative cells but biochemical characterizations have not been performed [220,410,414]. Therefore, additional studies are required to further dissect the physiological role of each Dps protein in filamentous heterocyst-forming cyanobacteria. Despite the expression evidences for the presence of four Dps proteins in heterocysts, the oxidative stress response network in heterocysts and diazotrophic filaments remains largely unknown. Thus, further investigations are needed to provide additional insights regarding this question and its importance in the diazotrophic physiology in heterocyst-forming cyanobacteria.

## 6. Energy Metabolism and Metabolic Networks in the Diazotrophic Filament

Heterocysts house three general metabolic pathways that are functional, namely the glycolysis pathway, the OPPP, and the Krebs cycle [415,416,417]. However, they are subjected to some adaptations that modify their metabolic flux to play different roles to that of vegetative cells. Therefore, such metabolic modifications are aimed at optimizing those pathways to the bioenergetics requirements in heterocysts.

### 6.1. Glycolysis and Oxidative Pentose Phosphate Pathways

**Figure 5:**
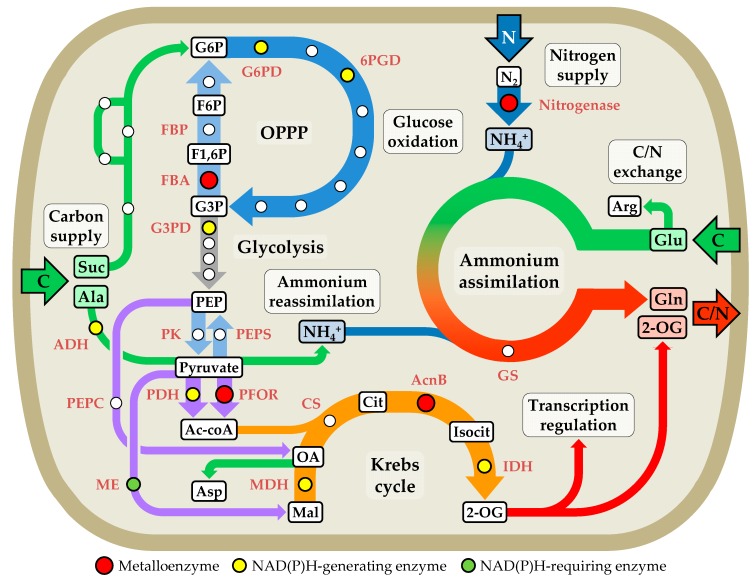
Main energy metabolism and carbon/nitrogen fluxes in heterocysts. The OPPP (oxidative pentose phosphate pathway) and the Krebs cycle are the major routes used by heterocysts to generate reducing equivalents, while glycolysis has a smaller contribution. Metalloenzymes (red circles) and enzymes catalyzing reactions that generate (yellow circles) or require (green circles) NAD(P)H are indicated. *Abbreviations for metabolites*: Suc, sucrose; G6P, glucose 6-phosphate; F6P, fructose 6-phosphate; F1,6P, fructose 1,6-bisphosphate; G3P, glyceraldehyde 3-phopshate; PEP, phosphoenolpyruvate; Ac-coA, acetyl-coA; Mal, malate; OA, oxaloacetate; Cit, citrate; Isocit, isocitrate; 2-OG, 2-oxoglutarate. *Abbreviations for enzymes*: G6PD, glucose 6-phosphate dehydrogenase; 6PGD, 6-phosphogluconate dehydrogenase; FBA, class-II fructose bisphosphate aldolase; FBP, fructose bisphosphatase; G3PD, glyceraldehyde 3-phosphate dehydrogenase; PK, pyruvate kinase; PEPS, PEP synthase; PDH, pyruvate dehydrogenase; PFOR, pyruvate:ferredoxin oxidoreductase; MDH, malate dehydrogenase; CS, citrate synthase; AcnB, aconitase, IDH, isocitrate dehydrogenase; GS, glutamine synthetase. Cyanophycin metabolism and the reported transfer to vegetative cells of the cyanophycin-derived dipeptide β-aspartyl-arginine [120] are not included in the scheme.

Heterocysts rely on energy provided by vegetative cells in the form of sucrose, since they cannot generate reducing equivalents through photosynthesis. This sugar is metabolized in heterocysts generating glucose-6 phosphate (G6P), which represents the major source of reducing equivalents in these cells and is subsequently degraded through the OPPP (Figure 5) [140,418,419]. The OPPP is the main energy metabolic pathway and the predominant route for the degradation of G6P in heterocysts, although the Krebs cycle and, to a lesser extent, the glycolysis pathway can also provide some reducing equivalents.

The initial OPPP enzymes are highly expressed in heterocysts, especially G6P dehydrogenase and phosphogluconate dehydrogenase, to ensure a preferential metabolic flux of G6P through this route rather than glycolysis [415,417,420,421]. These enzymes catalyze the oxidative decarboxylation of G6P, rendering two molecules of NADPH and ribulose 5-phosphate (R5P), which is metabolized then by isomerases, transketolases, and transaldolases to produce glyceraldehyde 3-phosphate (G3P) and G6P. Thus, the non-oxidative phase of the OPPP enables the regeneration of G6P to render more NADPH through the oxidative phase of the OPPP, whereas G3P is channeled to a middle step in the glycolysis pathway (Figure 5). In this metabolic step lies the metalloenzyme fructose-1,6-bisphosphate aldolase.

Fructose-1,6-bisphosphate aldolases catalyze the reversible cleavage and formation of fructose 1,6-bisphosphate (FBP) from glyceraldehyde 3-phosphate (G3P) and dihydroxyacetone phosphate (DHAP) and represent an important metabolic step in glycolysis, gluconeogenesis, and the reductive pentose phosphate cycle [422,423]. FBP aldolases are grouped into two different classes of enzymes which share no significant sequence identity, thus it is presumed that both classes appeared and evolved independently from each other [423,424]. Despite the fact that members of both classes display a similar structure, they use different enzymatic mechanisms [425]. Class-I FBP aldolases (FbaB), which depend on the formation of a substrate intermediate between a catalytic Lys residue and the substrate [426], are mainly found in eukaryotes and form homotetramers [427,428,429,430]. On the other hand, class-II FBP aldolases (FbaA or FBA) are metalloenzymes that require divalent cations, generally Zn^2+^ ions, to stabilize the catalytic substrate intermediate during the reaction [422,431]. They represent the major FBP aldolases in bacteria and form homodimers. Cyanobacteria contain proteins of both classes, but FbaA enzymes seem to be predominantly expressed [234,421,423,432]. In *Anabaena* sp. PCC 7120, *all3735* and *all4563* encode FbaB and FbaA, respectively [421,432], while ORFs (open reading frame) *Npun_R0192* and *Npun_F5584* encode FbaB and FbaA, respectively, in *N. punctiforme* ATCC 29133 [234]. 

FbaA members are arranged as homodimeric metalloenzymes, with each monomer displaying a conserved (α/β)_8_-barrel fold that contains an active-site Zn^2+^ ion and a non-catalytic Zn^2+^ ion nearby (Table 7) [422]. The active Zn^2+^ site is involved in the reaction catalysis and is localized at the C-terminal end of the barrel, within a substrate-binding pocket that is present in this region. This site is exposed to the solvent on the surface of the binding pocket and the catalytic Zn^2+^ ion is coordinated to three conserved His residues and further stabilized via interactions with the substrate to complete the coordination sphere (Figure 2M, right) [422]. In turn, the non-catalytic Zn^2+^ ion site is buried in the enzyme next to the catalytic site and the substrate-binding pocket. This second Zn^2+^ ion is coordinated to the side chains of one Asp and two Glu residues and a H_2_O molecule (Figure 2M, left) and plays an important structural role stabilizing the positions of two His residues involved in the coordination of the catalytic Zn^2+^ ion [422]. Thus, the second metal-binding site contributes to an appropriate arrangement and stabilization of the catalytic site [422,431,433].

Although the structure of FbaA from heterocyst-forming cyanobacteria has not been resolved, cyanobacterial FbaAs contain all metal ligands and are predicted to display the structure described above. However, some results in the unicellular cyanobacterium *Synechocystis* sp. PCC 6803 suggest that Co^2+^, rather than Zn^2+^, could be the preferred metal in cyanobacterial FbaA enzymes [423]. Nevertheless, this is not a strong indication, as Co^2+^-substituted enzymes often exhibit hyperactivity compared to their Zn^2+^-containing forms [434].

Heterocysts exhibit FBP aldolase activity [415] and seem to express the metal-dependent FbaA aldolase preferentially [421]. Although this enzyme is located in a central position in the metabolism of heterocysts, next to the OPPP-exit branch that supplies G3P to glycolysis, it does not seem to play an essential role in the regulation of both pathways, since its activity is lower in heterocysts than in vegetative cells [415]. On the contrary, the expression of the gluconeogenesis enzyme fructose 1,6-bisphosphatase is highly upregulated in heterocysts, whereas the abundance of the glycolytic 6-phosphofructokinase is low [265,420,421], suggesting that the upper half of the glycolysis pathway operates anabolically, as in gluconeogenesis, and regenerates G6P from the OPPP-derived G3P (Figure 5). This route seems to be predominant in heterocysts, creating a metabolic cycle between the OPPP and the upper half of the gluconeogenesis that ensures an efficient route to fully oxidize G6P via the OPPP for the generation of NADPH. These observations are supported by the role of G3P dehydrogenase in heterocysts. These cells express an NAD(H)- and an NADP(H)-dependent G3P dehydrogenase that exhibit different abundances and perform different metabolic functions [417,419,420,421]. The NAD(H)-dependent enzyme is the most abundant and, despite carrying out both reactions, preferentially catalyzes the gluconeogenesis reaction, while the NADP(H)-dependent enzyme preferentially carries out the glycolytic route generating NADPH but provides a lower contribution to the net reaction [419]. This also illustrates that heterocysts preferentially use NADH for the anabolic reaction, while NADPH is favored for Fd reduction and N_2_ fixation [419]. Thus, the presence of two enzymes enables heterocysts to use the OPPP-derived G3P preferentially for G6P regeneration, while the lower glycolysis pathway, despite being hampered, is not completely abolished and can generate pyruvate to some extent (Figure 5).

The catabolism of G3P generates phosphoenolpyruvate (PEP), which can provide the Krebs cycle with oxaloacetate through the enzyme PEP carboxylase (PEPC) present in heterocysts [416,421,435]. Moreover, the gene expression of PEPC is moderately upregulated in heterocysts [421]. This enzyme plays an important role in heterocysts supplying metabolites to feed the reductive branch of the Krebs cycle that, otherwise, would not enable the synthesis of citrate and, ultimately, 2-OG (Figure 5) [416]. Furthermore, oxaloacetate and Glu are also important precursors for the synthesis of Asp and Arg, respectively (Figure 5) [436]. Both amino acids are required to synthesize cyanophycin, which is a nitrogen-storage compound made of multi-L-arginyl-poly-L-aspartate where α-amino groups of Arg residues are linked to β-carboxyl groups of a poly-Asp backbone [120,437]. In turn, PEP can be also metabolized in heterocysts via the enzyme pyruvate kinase (PK) through an irreversible reaction that generates pyruvate (Figure 5) [220,416,421], which is the last metabolite of the glycolysis pathway.

### 6.2. Pyruvate Metabolism

Pyruvate is a central metabolite in the cellular metabolism, connecting the Krebs cycle and the glycolysis pathway. However, the latter can only provide pyruvate to a limited extent in heterocysts and an additional supply to cope with the high metabolic demands in these cells is required. Thus, vegetative cells in the diazotrophic filament transfer the amino acid Ala to heterocysts [438,439], where alanine dehydrogenase (Ald) is highly induced and catabolizes Ala into pyruvate, NADH, and NH_4_^+^ (Figure 5) [439]. As with NH_4_^+^ formed through nitrogenase, NH_4_^+^ produced by Ald is also assimilated by the enzyme GS into Gln, which is then transferred to vegetative cells.

Pyruvate is the direct substrate of important metabolites for the Krebs cycle in heterocysts: Acetyl-coA, phosphoenolpyruvate (PEP), and malate. Acetyl-coA is synthesized by the pyruvate dehydrogenase complex (PDH) [234,421,440] and the metalloenzyme PFOR [415,421], while PEP could be generated by the enzyme PEP synthase (PEPS; Figure 5) [421,441,442]. Interestingly, the genome of *Anabaena* sp. PCC 7120 bears five genes that may encode PEPSs (*all0635*, *alr3146*-*alr3147,* and *alr3397*) and an enzyme similar to PEPSs (*all2509*) [421,442,443,444]. Some of these genes are strongly (*all0635*) or moderately (*alr3146*-*alr3147*) downregulated in heterocysts, whereas only *all2509* seems to be somewhat upregulated [421]. In turn, newly synthesized PEP can be converted into oxaloacetate via the enzyme PEPC, providing carbon skeletons to the reductive branch of the Krebs cycle (Figure 5) [416]. PEPS has been shown to enable *E. coli* growth solely on pyruvate, Ala, or lactate [441]. Thus, this metabolic route could also allow heterocysts to metabolize Ala from vegetative cells and provide carbon compounds for the biosynthesis of 2-OG and the cyanophycin-precursor Asp [416,442,445]. Pyruvate could also provide the reductive branch of the Krebs cycle with malate through the malic enzyme (ME; Figure 5), which is present in heterocysts and whose gene (*alr4596*) expression is upregulated [416,421]. This enzyme can catalyze the reversible interconversion of malate and pyruvate [416,442,446]. Interestingly, heterocysts exhibit high NAD^+^- and NADP^+^-dependent malate dehydrogenase (MDH) activities (although the former is predominant) that could rapidly covert malate into oxaloacetate [416,421]. Thus, heterocysts seem to contain all the enzymes required for the conversion of pyruvate and PEP into 2-OG and oxaloacetate. Acteyl-coA, oxaloacetate, and potentially malate represent important input metabolites in the Krebs cycle that contribute to the production of energy, reducing equivalents, and biosynthetic precursors. However, despite the high importance of pyruvate metabolism in heterocysts, little is known about its metabolic flux and regulation.

The enzyme PFOR catalyzes the oxidative cleavage of pyruvate and coenzyme A to acetyl-coA and CO_2_ with concomitant reduction of two molecules of Fd or one molecule of flavodoxin [440,447,448,449], rather than one NADH molecule as catalyzed by the enzyme PDH [448]. PFORs are O_2_-sensitive metalloenzymes widely distributed in archaea and bacteria, being present mainly in strict anaerobes but also in some aerobes, including cyanobacteria [440,447,450]. The electron acceptor is usually either Fd or flavodoxin [450], although the Fe–S protein rubredoxin can be also an alternative electron acceptor [451]. However, unlike the PDH complex, PFOR can catalyze the reverse reaction carrying out a reductive carboxylation of acetyl-coA into pyruvate [447,452]. Despite cyanobacterial PFORs are more closely related to the enzymes found in anaerobes than to those in aerobes, they are expressed under oxic conditions in cyanobacteria [152,447], although the enzyme is also inactivated by O_2_ [415].

The overall structure of PFOR enzymes is also rather variable. Most bacterial members form homodimers (A_2_), yet dimers of heterodimers (a_2_b_2_) are also found in a few cases, whereas PFORs in archaea are heterotetramers (αβγδ), which are considered to resemble the ancestral protein arrangement [450,453]. Despite the differences in subunit composition, they share a similar structure and topology. Therefore, subunits A contain domains homologous to the four α, β, γ, and δ subunits of archaeal members, while ab dimers exhibit domains homologues to α, β, and γ subunits, but lack a domain similar to subunits δ [450,453]. 

In archaeal heterotetrameric PFORs, the subunit δ harbors the binding motifs to coordinate two Fd-like [4Fe–4S] clusters and the subunit β contains a conserved binding site for a thiamine pyrophosphate cofactor and the Cys residues to coordinate one [4Fe–4S] cluster, while subunits α and γ are involved in catalytic and structural roles [450,453]. As in heterotetrameric PFORs, bacterial homodimeric enzymes dispose the same cofactors in a similar arrangement per monomer. Although the structure of PFORs from heterocyst-forming cyanobacteria has not been resolved, PFORs from this group of cyanobacteria exhibit sequence similarity to that of bacterial homologs and are predicted to contain the same metal clusters and the thiamine pyrophosphate cofactor [447,453]. Thiamine pyrophosphate and one [4Fe–4S] cluster are buried in close proximity within the protein near the substrate binding site [450]. This proximal Fe–S cluster is coordinated to four Cys residues located in an atypical Cys-containing sequence motif with no resemblance to the usual Fd-binding motifs [452]. The other two [4Fe–4S] clusters, which are termed medial and distal clusters according to their distance to the thiamine pyrophosphate cofactor, lead toward the enzyme surface and are part of a Fd-like domain, where both clusters are coordinated to standard Fd binding motifs via four Cys residues each [450,452,454]. The sequential arrangement of the three Fe–S clusters at equidistant positions between the thiamine pyrophosphate and the enzyme surface provides an electron transfer pathway from the substrate binding site to the redox partners Fd or flavodoxin, which interact with the enzyme through a binding pocket provided by the Fd-like domain [450,452]. Interestingly, this domain displays similarity to the acceptor side of PSI, which harbors two [4Fe–4S] clusters and also binds Fd and flavodoxin [449,452,455].

In heterocyst-forming cyanobacteria, PFOR (NifJ, Alr2803 in *Anabaena* sp. PCC 7120; Table 7) is expressed in heterocysts and catalyze the reduction of Fd from pyruvate for N_2_ fixation, although the physiological role of this enzyme in the diazotrophic filament is not totally understood [415,421]. This reaction could have an important contribution in Fd reduction for N_2_ fixation, since heterocysts have a continuous supply of pyruvate from vegetative cells in the form of Ala [438,439]. Moreover, as PFORs are O_2_-sensintive enzymes, heterocysts could represent an appropriate low-O_2_ environment for NifJ [447]. Interestingly, the enzyme PFOR can also reduce flavodoxin, which effectively transfers electrons to nitrogenase [152,447]. However, despite the fact that flavodoxins have been considered to be expressed only in Fe-limited conditions in cyanobacteria, some results indicate that they may be more frequent in Fe-repleted conditions that originally thought [152,447]. In this context, the presence of highly-induced genes in heterocysts encoding flavodoxin and flavoproteins [421], which have not been extensively explored in Fe-repleted conditions, suggests that a Fd-independent pathway could deliver electrons to nitrogenase and play an important role in the physiology and metabolism of these specialized cells in diazotrophic filaments. Therefore, the generation of mutants lacking FdxH and PFOR could contribute to elucidate such electron pathways. However, other additional unknown pathways could also provide electrons to nitrogenase and overlap functions in heterocysts, making the characterization of alternative routes more difficult.

### 6.3. Krebs Cycle

The Krebs cycle in heterocysts is aimed at metabolizing acetyl-coA and oxaloacetate to generate 2-OG, a pathway that also provides reducing equivalents in the form of NADPH via the isocitrate dehydrogenase (Figure 5), which exhibits a high activity in heterocysts [152,270,415]. In this biosynthesis process of 2-OG, the metalloenzyme aconitase, also known as aconitate hydratase, is essential.

Aconitases are monomeric proteins that catalyze the reversible interconversion of citrate and isocitrate via cis-aconitate in the Krebs cycle [456]. The ubiquitous aconitase superfamily is classified in various phylogenetic groups, two of which include bacterial aconitases. One group includes aconitases A (AcnA), which are similar to eukaryotic aconitases and are expressed under stress conditions, while the other group comprises aconitases B (AcnB), which display a structure unique to bacteria and play an essential role as the main Krebs cycle enzyme in both vegetative cells and heterocysts in heterocyst-forming cyanobacteria [415,457,458].

Whereas most Fe–S proteins are involved in redox processes and function as electron carriers, aconitases contain a unique catalytic [4Fe–4S] cluster that reacts directly with the substrate citrate. This metal cluster is located in the substrate binding site and coordinated only to three Cys residues, while one of the Fe atoms is exposed to the solvent and coordinated to a molecule of H_2_O (Figure 2N) [458]. This non-liganded Fe atom plays an essential role in the citrate–isocitrate isomerization reaction binding citrate and promoting the removal of a proton and a hydroxyl group, a step that generates the cis-aconitate intermediate, followed by rehydration of cis-aconitate to form isocitrate [238,459]. This labile Fe atom is easily oxidized and released as Fe^2+^ in oxidizing conditions and in the presence of ROS, generating an enzymatically inactive [3Fe–4S] cluster [348,460]. Importantly, Fe^2+^ ions released in this process can exacerbate the oxidative stress and ROS generation via Fenton chemistry (see Section 5). Moreover, the inactive [3Fe–4S] cluster can be further degraded, turning AcnB metalloenzymes into apoenzymes [458]. Metal-cluster-free AcnB aconitases can also bind to specific sequences in the 3’-UTR (untranslated) regions of *acnB* mRNA, promoting the synthesis of AcnB under stress conditions that deplete the metal-cluster-bound active form, such as Fe starvation or oxidative stress. Therefore, enzymatically inactive aconitases can play a dual role as enzymes and post-transcriptional regulators [458]. Moreover, aconitases in *E. coli* are also involved in regulating the synthesis of SOD enzymes [461], a mechanism that could be functional in cyanobacteria. However, little is known about such mechanisms in these organisms and, thus, there is still much to learn about the importance of the regulatory roles of aconitases and the cellular components associated with their regulatory functions in cyanobacteria.

In heterocysts, the metalloenzyme AcnB (All1267 in *Anabaena* sp. PCC 7120) operates in the Krebs cycle as in vegetative cells to ultimately generate 2-OG (Figure 5; Table 7). However, since the enzyme GOGAT is not expressed in heterocysts unlike in vegetative cells [141], 2-OG cannot be metabolized into Glu and, thus, accumulates in the cell. This high concentration serves a dual role in heterocysts. Firstly, 2-OG plays a regulatory role in gene expression by activating NtcA (Figure 5) [462], which is essential to induce the expression of important genes for the physiology, bioenergetics, and metabolism of heterocysts [68,143]. Secondly, 2-OG excess may be transferred to vegetative cells simultaneously with Gln (Figure 5) [442,445], where a complete GS–GOGAT cycle is carried out to form two molecules of Glu [141]. In turn, Glu is returned to heterocysts, where the enzyme GS catalyzes its condensation with NH_4_^+^ to regenerate Gln for vegetative cells (Figure 5) [442,445]. The periplasmic space and septosomes connecting cells could play an important role in the transfer of molecules between heterocysts and vegetative cells in the diazotrophic filament, although the mechanisms of transfer and their regulation are not totally understood yet [75,463]. Thus, this is a fundamental question that has to be addressed to completely understand the diazotrophic physiology of heterocyst-forming cyanobacteria. 

## 7. Concluding Remarks

Photoautotrophic bacteria exhibit higher transition metal demands than other microorganisms. These raised demands are partially attributed to the photosynthetic apparatus. However, filamentous heterocyst-forming cyanobacteria are able to transform vegetative cells into heterocysts (Section 2). These cells are specialized in N_2_ fixation and, thus, they require a massive reprogramming of the cellular ultrastructure and proteome to efficiently carry out this function. This also includes a further increase of transition metal requirements involved as cofactors in a wide range of metalloproteins, since the chemical composition of amino acid residues does not provide the capability to perform all chemical reactions required to maintain key cellular functions in heterocysts (Section 1). It has been proposed that the requirement of particular metals for the function of numerous metalloproteins is related to the bioavailability of metals at the time such metalloproteins emerged and evolved [464,465]. This partially explains the high integration of transition metals into the catalytic centers of proteins prone to oxidation (and inactivation), despite the fact that the evolution of cyanobacteria took place simultaneously with the origin of atmospheric O_2_ [272]. Moreover, it is currently considered that heterocysts arose and evolved to protect the O_2_-sensitive process of N_2_ fixation as a consequence of the cyanobacterial success in O_2_ photosynthesis [466,467]. This further explains the recycling of many metal-containing proteins and complexes from vegetative cells in heterocysts, e.g., in energy generation (Section 4), metabolic networks (Section 6), or transcriptional and translational regulation (not discussed in this review). Nevertheless, additional metalloenzymes have evolved during the course of heterocyst evolution, particularly to remove O_2_ and radical oxygen species (Section 4 and Section 5) that, otherwise, would inhibit metalloenzymes involved in the N_2_-fixation apparatus (Section 3). The wealth of metalloproteins involved in heterocyst formation and function renders this cell type highly sensitive to low environmental metal quota. At present, seven *d*-block metals, namely Fe, Cu, Mo, Ni, Mn, V, and Zn, are found as central components of metalloproteins required for heterocyst function. Thus, the extreme metal requirements of heterocysts far exceed those of vegetative cells and most groups of microorganisms, making heterocysts highly metal-dependent and prolific cells in the management and use of transition metals. While the physiological requirements on metals for the heterocyst function has been explored under limiting and toxic metal levels, future research on the protein composition and the biochemical pathways of this special bacterial cell might uncover not only additional metal-containing proteins but also novel metalloproteins dependent on other *d*-block metals.

## Figures and Tables

**Table 1:** Major features of metalloproteins in organisms [4,15,21,25,26,42,43,44,45].

Metal	Major Catalytic and Structural Roles
Alkali	
Na^+^	Charge carrier; ionic gradient generation
K^+^	Charge carrier; ionic gradient generation; enzyme activation
Alkaline earth	
Mg^2+^	Phosphoryl transfer; soft-bond hydrolysis; enzyme activation; *chlorophyll* ^‡^
Ca^2+^	Cell signaling and structural trigger; acid-base catalysis; enzyme activation
Transition ^†^	
V	N_2_ activation; *e*^−^ transfer
Cr	Cell signaling and potential structural trigger
Mo	N_2_ activation; *e*^−^ transfer; hydroxylation (H_2_O)
W	*e*^−^ transfer; hydroxylation (H_2_O)
Mn	O_2_ evolution (H_2_O); *e*^−^ transfer; acid-base catalysis; hydrolysis; Mg^2+^ surrogate
Fe	O_2_ activation; *e*^−^ transfer; acid-base catalysis; hydroxylation (O_2_); H transfer ^§^; *heme* ^‡^
Co	Methyl transfer; isomerization catalysis; *e*^−^ transfer; H transfer ^§^; *cobalamin* ^‡^
Ni	H_2_, CH_4_ and CO activation; *e*^−^ transfer; acid-base catalysis; H transfer ^§^; *cofactor F_430_* ^‡^
Cu	O_2_ activation; *e*^−^ transfer; acid-base catalysis; hydroxylation (O_2_); H transfer ^§^
Group 12	
Zn^2+^	Acid-base catalysis; strong-bond hydrolysis; enzymes in all EC classes; Zn fingers
Cd^2+^	Acid-base catalysis in carbonic anhydrase in diatoms

^†^ Charge is not given for transition metals as it varies with the oxidation state during enzymatic catalysis. ^‡^ Mg^2+^-, Fe-, Co-, and Ni-containing cyclic tetrapyrroles are indicated in *italics*. ^§^ H transfer stands for hydrogen transfer.

**Table 2:** Heterocyst-specific nitrogenases and hydrogenases.

Enzyme	Subunit(s)	Protein(s)	Cofactor(s)	Metal ^†^
Mo nitrogenase	Dinitrogenase reductase	NifH	Mg^2+^-ATP	1 Mg^2+^
	(Fe protein; subunit γ)		[4Fe–4S] cluster	4 Fe
	Dinitrogenase	NifDK	[8Fe–7S] P cluster	15 Fe
	(MoFe protein; subunits αβ)		[Mo–7Fe–9S–C–*h* ^§^] FeMo-co	1 Mo
V nitrogenase	Dinitrogenase reductase	VnfH	Mg^2+^-ATP	1 Mg^2+^
	(Fe protein; subunit γ)		[4Fe–4S] cluster	4 Fe
	Dinitrogenase	VnfDKG	[8Fe–7S] P cluster	15 Fe
	(VFe protein; subunits αβδ)		[V–7Fe–8S–C-*h* ^§^] FeV-co	1 V
Ni–Fe uptake hydrogenase	Hydrogenase (subunit α)	HupL	[Ni–Fe] cluster	1 Ni 1 Fe
	Hydrogenase (subunit β)	HupS	[3Fe–4S] cluster 2 × [4Fe–4S] clusters	11 Fe
Ni–Fe bidirectional hydrogenase	Hydrogenase (subunit α)	HoxH	[Ni–Fe] cluster	1 Ni 1 Fe
	Hydrogenase (subunit β)	HoxY	[4Fe–4S] cluster	4 Fe
	Diaphorase (large subunit)	HoxF	[2Fe–2S] cluster	6 Fe
			[4Fe–4S] cluster	
			NAD(P)H/NAD(P)^+^	–
			FMN	–
	Diaphorase (small subunit)	HoxU	[2Fe–2S] cluster	14 Fe ^‡^
			3 × [4Fe–4S] clusters ^‡^	
	Diaphorase (bridging subunit)	HoxE	[2Fe–2S] cluster	2 Fe

^†^ Charge is not given for transition metals as it varies with the oxidation state during enzymatic catalysis. ^‡^ The configuration of one Fe–S cluster is uncertain and might be [4Fe–4S] or [3Fe–4S]. HoxU contains 14 or 13 atoms of Fe. ^§^ *h* stands for homocitrate.

**Table 3:** Metalloproteins in the photosynthetic electron transport chain in heterocysts.

Complex	Subunit(s)	Protein(s)	Cofactor(s)	Metal ^†^
Photosystem I	–	PsaABJKLMX, RC ^§^	Chlorophyll *a*	96 Mg^2+^
	Fe–S protein	PsaAB	[4Fe–4S] cluster F_X_	4 Fe
	Fe–S protein	PsaC	[4Fe–4S] cluster F_A_	8 Fe
			[4Fe–4S] cluster F_B_	
Cytochrome *b*_6_*f*	Subunit IV	suIV	Chlorophyll *a*	1 Mg^2+^
	Cytochrome *b*_6_	PetBD	Heme *b*_p_	3 Fe
			Heme *b*_n_	
			Heme *x* (or *c*_n_)	
	Cytochrome *f*	PetA	Heme *f*	1 Fe
	Rieske protein	PetC	[2Fe–2S] Rieske cofactor	2 Fe
Photosystem II ^‡^	–	PsbABCD	Chlorophyll *a*	36 Mg^2+^
	D1 protein	PsbA	H_2_O-splitting Mn cluster	4 Mn
	D1/D2 proteins	PsbAD	Non-heme Fe center	1 Fe
	Cytochrome *b*_559_	PsbEF	Heme *b*_559_	1 Fe
	Cytochrome *c*_550_	PsbV	Heme *c*_550_	1 Fe
Photosystem II inactivation	FtsH protease	FtsH	Mg^2+^-ATP	1 Mg^2+^
			Zn^2+^ center	1 Zn^2+^
Soluble electron Carriers	Plastocyanin	PetE	Cu center	1 Cu
	Cytochrome *c*_6_	PetJ	Heme *c*_553_	1 Fe
	Ferredoxin	FdxH	[2Fe–2S] cluster	2 Fe
	Ferredoxin	FdxN	2 × [4Fe–4S] clusters	8 Fe

^†^ Charge is not given for transition metals as it varies with the oxidation state during electron transfer. ^‡^ Photosystem II is inactivated in heterocysts. ^§^ RC stands for reaction center.

**Table 4:** Metalloproteins in the respiratory electron transport chain in heterocysts.

Family	Complex	Protein	Cofactor(s)	Metal ^†^
NAD(P)H dehydrogenases	Type-1 NADP(H) dehydrogenase NDH-1	NdhI	2 × [4Fe–4S] clusters N6a, N6b	8 Fe
		NdhK	[4Fe–4S] cluster N2	4 Fe
Heme-copper oxidases	*aa*_3_-type cytochrome *c* oxidase Cox2	CoxA2	Cu center Cu_B_	1 Cu
			Heme *a*_3_	2 Fe
			Heme *a*	
		CoxB2	Cu–Cu center Cu_A_	2 Cu
	*bo*_3_-type quinol oxidase Qox (ARTO; Cox3)	QoxA (CoxA3 ^§^)	Cu center Cu_B_	1 Cu
			Heme *o*_3_	2 Fe
			Heme *b*	
Quinol oxidases	Cytochrome *bd* quinol oxidase	CydA (All4024 ^§^)	Heme *b*_595_	3 Fe
			Heme *d*	
			Heme *b*_558_	
Alternative oxidases	Plastoquinol terminal oxidase	PTOX ^‡^ (All2096 ^§^)	Non-heme Fe–Fe center	2 Fe

^†^ Charge is not given for transition metals as it varies with the oxidation state during electron transfer. ^‡^ The presence of PTOX in heterocysts has to be confirmed experimentally. ^§^ Specific examples in the strain *Anabaena* sp. PCC 7120.

**Table 5:** Flavodiiron proteins in heterocysts.

Family	Group	Subunit	Protein	Cofactor(s)	Metal ^†^
Class-C flavodiiron proteins	Type 3	Flavodiiron protein (flavoprotein)	Flv1B (All0177 ^§^)	Non-heme Fe–Fe center	2 Fe
				NAD(P)H ^‡^	–
				FMN	–
	Type 1	Flavodiiron protein (flavoprotein)	Flv3B (All0178 ^§^)	Non-heme Fe–Fe center	2 Fe
				NAD(P)H ^‡^	–
				FMN	–

^†^ Charge is not given for Fe as it varies with the oxidation state during electron transfer. ^‡^ Heterocyst-specific flavodiiron proteins might use ferredoxin [257]. ^§^ Proteins in *Anabaena* sp. PCC 7120 [330].

**Table 6:** Metalloproteins involved in oxidative stress management in heterocysts.

Family	Enzyme/Complex	Protein(s)	Cofactor(s)	Metal ^†^
Superoxide dismutases	Mn–SOD	SodA	Mn center	1 Mn
	Fe–SOD	SodB	Non-heme Fe center	1 Fe
Non-heme peroxidases (ferritin-like superfamily)	Rubrerythrin	RbrA (Alr1174 ^§^)	Non-heme Fe–Fe center	3 Fe
			[Fe–4S] cluster	
	Dps proteins	Dps1 (All0458 ^§^)	Non-heme Fe–Fe center	2 Fe ^‡^
		Dps2 (All4145 ^§^)	Non-heme Fe–Fe center	2 Fe ^‡^
		Dps3 (All1173 ^§^)	Non-heme Fe–Fe center	2 Fe ^‡^
		Dps4 (Alr3808 ^§^)	Non-heme Fe–Fe center	2 Fe ^‡^

^†^ Charge is not given for transition metals as it varies with the oxidation state during enzymatic catalysis. ^‡^ Atoms of Fe per ferroxidase center. Dps protein complexes harbor 12 catalytic Fe–Fe centers [388]. ^§^ Specific examples in the strain *Anabaena* sp. PCC 7120.

**Table 7:** Metalloenzymes involved in energy metabolism in heterocysts.

Pathway	Enzyme	Protein(s)	Cofactor(s)	Metal ^†^
Glycolysis	Fructose-1,6-bisphosphate aldolase	FbaA (FBA; All4563 ^‡^)	Catalytic Zn^2+^ center	1 Zn^2+^
			Structural Zn^2+^ center	1 Zn^2+^
Pyruvate metabolism	Pyruvate:ferredoxin (flavodoxin) oxidoreductase	NifJ (PFOR; Alr2803 ^‡^)	3 × [4Fe–4S] clusters	12 Fe
			Thiamine pyrophosphate	–
Krebs cycle	Aconitase (aconitate hydratase)	AcnB (All1267 ^‡^)	Catalytic [4Fe–4S] cluster	4 Fe

^†^ Charge is not given for Fe as it varies with the oxidation state during enzymatic catalysis. ^‡^ Specific examples in the strain *Anabaena* sp. PCC 7120.
